# Investigating behavioral drivers of seasonal *Shiga-Toxigenic Escherichia Coli (STEC)* patterns in grazing cattle using an agent-based model

**DOI:** 10.1371/journal.pone.0205418

**Published:** 2018-10-10

**Authors:** Daniel E. Dawson, Jocelyn H. Keung, Monica G. Napoles, Michael R. Vella, Shi Chen, Michael W. Sanderson, Cristina Lanzas

**Affiliations:** 1 Department of Pathobiology and Population Health, College of Veterinary Medicine, North Carolina State University, Raleigh, North Carolina, United States of America; 2 National Institute for Mathematical and Biological Synthesis (NIMBioS), Knoxville, Tennessee, United States of America; 3 Department of Public Health Sciences, College of Health and Human Services, University of North Carolina Charlotte, Charlotte, North Carolina, United States of America; 4 Center for Outcomes Research and Epidemiology, Department of Diagnostic Medicine and Pathobiology, College of Veterinary Medicine, Kansas State University, Manhattan, Kansas, United States of America; The University of Sydney, AUSTRALIA

## Abstract

The causes of seasonal variability in pathogen transmission are not well understood, and have not been comprehensively investigated. In an example for enteric pathogens, incidence of *Escherichia coli* O157 (STEC) colonization in cattle is consistently higher during warmer months compared to cooler months in various cattle production systems. However, actual mechanisms for this seasonality remain elusive. In addition, the influence of host (cattle) behavior on this pattern has not been thoroughly considered. To that end, we constructed a spatially explicit agent-based model that accounted for the effect of temperature fluctuations on cattle behavior (direct contact among cattle and indirect between cattle and environment), as well as its effect on pathogen survival in the environment. We then simulated the model in a factorial approach to evaluate the hypothesis that temperature fluctuations can lead to seasonal STEC transmission dynamics by influencing cattle aggregation, grazing, and drinking behaviors. Simulation results showed that higher temperatures increased the frequency at which cattle aggregated under shade in pasture, resulting in increased direct contact and transmission of STEC between individual cattle, and hence higher incidence over model simulations in the warm season. In contrast, increased drinking behavior during warm season was not an important transmission pathway. Although sensitivity analyses suggested that the relative importance of direct vs. indirect (environmental) pathways depend to upon model parameterization, model simulations indicated that factors influencing cattle aggregation, such as temperature, were likely strong drivers of transmission dynamics of enteric pathogens.

## Introduction

Recurrent seasonality in disease incidence is common in infectious diseases [1,2]. Generally, incidence of bacterial enteric pathogens is greater during warmer months in both human and animal populations [3], but respiratory and viral enteric pathogens incidence peaks during colder months [4]. Seasonality is caused by various mechanisms. For example, environmental factors can influence pathogen abundance, survival and virulence or influence host susceptibility [1,2], and changes in host behavior and aggregation in different seasons can alter contact patterns, leading to different transmission dynamics [1,2]. Investigating mechanisms underlying disease seasonality is challenging because different mechanisms often interact in multiple causal pathways [5]. In infectious disease transmission models, seasonality is often included phenomenologically (e.g. sinusoidal function)[1] to evaluate its implications on disease dynamics, but not the causes leading to seasonality fluctuations. Hence, mechanistic, system-based methods are necessary to identify and quantify the contribution of different transmission drivers and causal pathways involved in seasonality of disease incidence [6]. Despite the need for such approaches to address environmental drivers of disease, the application of system-based methodologies such as agent-based modeling (ABM) has been limited.

Shiga-toxigenic *Escherichia coli* (STEC) are human zoonotic enteric pathogens that can cause severe illnesses including hemorrhagic colitis and hemolytic uremic syndrome [7]. Healthy cattle and their environment are the main reservoirs for STEC [8]. For humans, the most common source of exposure and subsequent infection is food and water contaminated by cattle feces containing STEC [8,9]. Understanding the biological and environmental factors leading to the persistence and transmission of STEC among cattle populations is necessary to design and improve control strategies. One of the most consistent patterns observed in STEC epidemiology is the strong seasonal pattern of fecal STEC shedding and increased prevalence during the warmer months in all cattle production systems, including grazing systems [10–13]. Multiple potential mechanisms have been proposed, but their relevance in contributing to the seasonal variations in STEC prevalence remain inconclusive (Alam and Zurek, 2004 [11,14]. Proposed mechanisms include enhanced transmission mediated by water and flies at higher temperature [11], and increased susceptibility linked to hormonal fluctuations associated with changes in day length [15]. Overall, evidence is limited for these proposed mechanisms [11].

**Fig 1:**
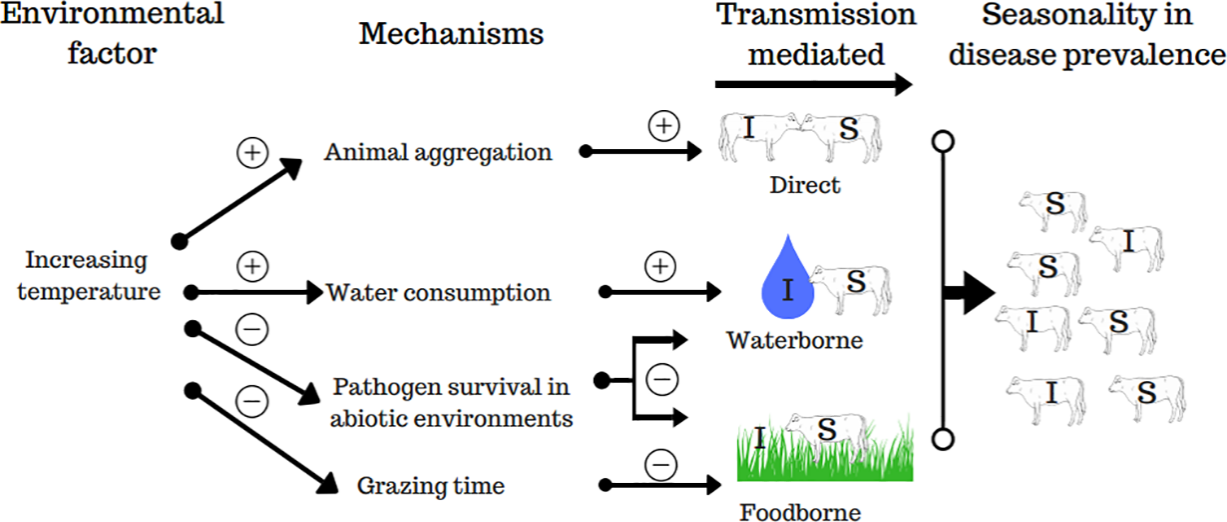
Seasonal temperature drives different behavioral mechanisms of STEC dynamics in cattle. Schematic of the transmission-based mechanisms by which high temperatures influence disease prevalence. Signs at arrows indicate the polarity of the relationship.

One possible mechanism that has received less attention is behavior changes in cattle in response to temperature fluctuations. There are several pathways of STEC transmission in cattle, including both direct host-to-host transmission and indirect environment-to-host transmission. Mutual grooming, aerosols, and contact with excretions (feces, urine) on the bodies of other individuals are important sources of direct transmission [16,17]. Consumption of contaminated water and food are potential sources of indirect transmission[18,19]. As temperature increases, cattle spend less time grazing and more time resting under the shade as a group to seek relief from heat stress [20–22]. In addition to escaping heat from solar radiation, feeding is reduced to decrease the heat associated with feed fermentation [23], with thresholds for reductions in feeding activity reported between 25 and 30°C [23,24]. In addition, cattle tend to consume more water as temperature increases [25]. For example, temperature increasing from 10°C to 32°C leads to 2.5 times more water consumption by calves [26]. This tends to occur via drinking larger volumes of water during each drinking, but not necessarily through more frequent drinking (MS, personal observation). These behavior changes are highly interrelated and can shape cattle exposure to pathogens and subsequent disease transmission (Fig 1).

Computational models are important tools to study complicated and dynamic systems. Agent-based models (ABM) provides a flexible framework to investigate relationships between observed patterns and hypothesized mechanisms underlying these patterns in complex ecological and epidemiological systems [27,28]. Agent-based models are particularly useful in linking transmission drivers and pathways with epidemiological patterns [29]. Furthermore, spatially explicit ABMs allow for the explicit consideration of spatial and temporal heterogeneity in host behavior as well as pathogen distribution in the environment, thus unifying the epidemiological triad: host, pathogen, and environment [18,30–32]. Therefore, to investigate mechanisms underlying seasonality in STEC incidence in grazing cattle, we developed a spatially explicit ABM for the transmission of STEC that incorporates varying mechanisms linking temperature and STEC transmission.

In this study, we aimed to evaluate how changes in animal behavior in response to temperature may influence the transmission patterns and the prevalence of the pathogen in the population of cattle in a pasture. In particular, we used the model to investigate two mechanisms by which temperature is hypothesized to influence transmission of STEC (Fig 1), including 1) by affecting the relative amount of time spent engaged in different activities (i.e., grazing vs. resting), and 2) by influencing the volume of water consumed by cattle.

## Materials and methods

### Model overview

We constructed a stochastic, spatially explicit agent-based model (ABM) to simulate transmission of STEC among grazing cattle. The model was written and executed in NetLogo 5.3.1 [33], an open-source agent-based modeling software. The model scope was a group of grazing beef cattle in an intensively managed pasture. A detailed model description in accordance with standard ODD (Overview, Design concepts, Details) protocol for individual- and agent-based models [34] is provided in S1 Text. We provide a brief overview below.

The purpose of this model was to quantify how temperature fluctuation caused changes in STEC incidence among grazing cattle by influencing diurnal behavior patterns. The model represented a 20-acre typical pasture consisting of patches (3.6 m^2^ (i.e., 1.9 x 1.9 m)), including 19 acres of a 80%/20% mixture of edible grass and inedible weeds, a 1-acre large pond with a constant depth of 0.5 m, and 5 trees that each provided a 4–patch radius (R = 7.6 m) of shade. See supplemental information (S1 Text) for a graphical representation of the model environment. The model simulated a closed cattle population (N = 25) as it engaged in different distinct activities throughout a model day (grazing, resting, drinking, sleeping). How cattle participated in these activities was influenced by the social state of an individual (dominant or subordinate), air temperature, and in the case of grazing, grass presence and length. In the first case, a single dominant individual influenced the movements of subordinate individuals during drinking and resting behaviors. In the second case, air temperature was included as an input variable (supplied via an external data file), and explicitly influenced aspects of cattle behaviors. In particular, the amount of time spent grazing versus resting was reduced, and the volume of drinking was increased with increased temperature. A temperature threshold also determined resting behavior, with temperatures above the threshold resulting in resting in groups under trees, while temperatures below the threshold resulted in cattle resting in the open field. Finally, grass grew at a constant rate over the course of the simulation to a maximum height, and was reduced in length by grazing to a minimum height, at which point it could not be grazed until it regrew.

**Fig 2:**
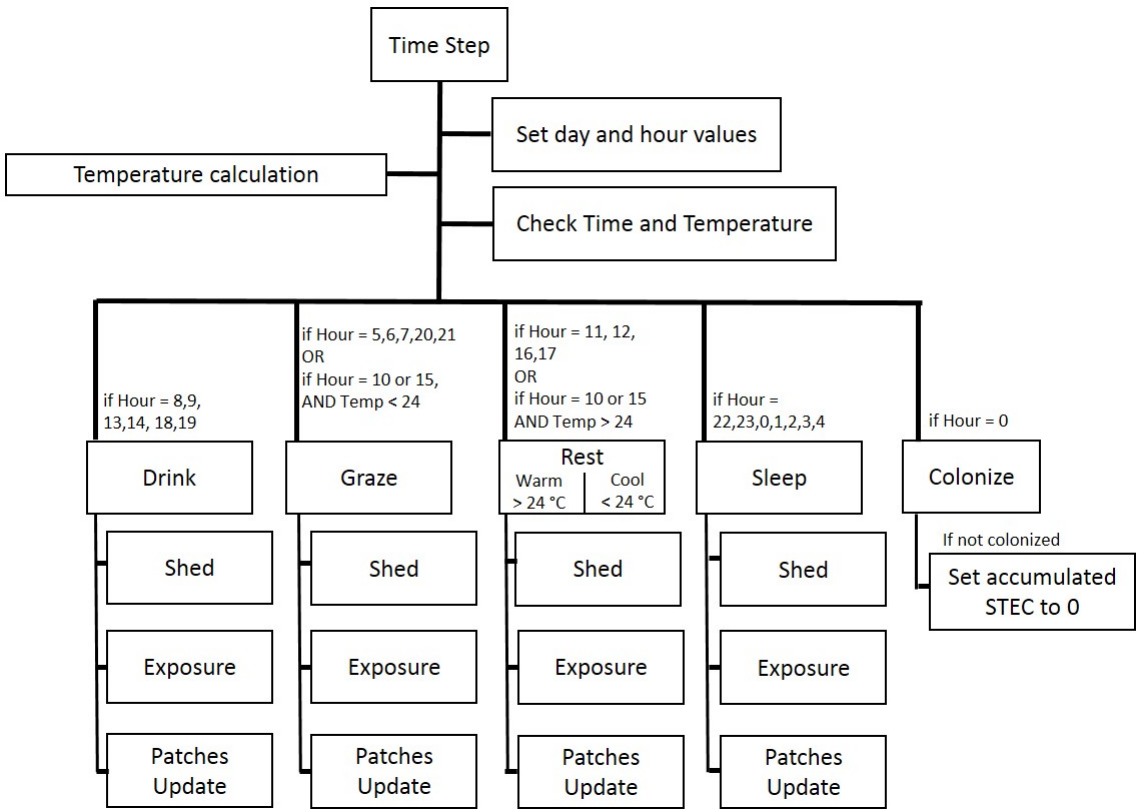
Schematic of time-step model operations and daily schedule of cattle activities. Model schedule and process order. Each box represents a sub-model. Model processes were determined by time of day (hour) and temperature. On a daily basis, cattle sleep, graze, drink, and rest. At each time step (10 minutes), cattle carry out the actions of the activity, have the opportunity to shed and be exposed to STEC, and patches update to reflect concentrations of STEC or grass height. At Hour 0 of each day, cattle are probabilistically colonized depending on the accumulated STEC from the previous day. Following the execution of the colonization sub-model, all accumulated STEC are reset to 0 in un-colonized cattle.

All major cattle activities recurred on an hourly schedule that repeated each model day. Sub-models governed stochastic cattle movements and transmission dynamics occurring during these activities on a 10-minute time-step. Simple rules governed daily animal activities and movements to generate realistic patterns of animal aggregation and fecal-pat distribution in the model environment. Some rules were derived from direct field observation of grazing cattle at the East Tennessee Research and Education Center—Blount Unit in August 2013 while others were based on existing literature on the topic. The schedule of the model, including the sequence of actions and their corresponding sub-models, is shown in Fig 2 and described in greater detail in S1 Text.

Superimposed on cattle activity was a Susceptible-Exposed-Infected-Recovered (SEIR)-type transmission model that simulated the transmission dynamics of STEC between cattle, and between cattle and the environment. Cattle could take one of 4 epidemiological states, including susceptible, colonized and in a latent period (without shedding), colonized and shedding, and partially susceptible (after becoming colonized once). Colonized cattle shed STEC in their feces, and susceptible cattle could become colonized through the daily accumulation of colony forming units (CFU’s) of STEC via direct contact with colonized individuals, through eating contaminated graze, or through drinking contaminated water.

To gain STEC through direct contact, susceptible cattle needed to come within a proximity threshold (*ddt****)*** of a colonized, shedding individual, at which point a quantity of colony forming units (CFU) of STEC was randomly drawn from a Poisson-lognormal distribution (S1 Text). Shedding individuals were assumed to shed a constant amount of CFU’s per fecal pat over the entire infectious period. To gain STEC through drinking, cattle had to drink from a water patch contaminated with infectious feces. The CFU’s up-taken during a drinking session was proportional to the concentration of STEC in the water patch (total CFU’s deposited/volume of patch, assuming homogenous distribution in patch), and the volume of water consumed. The volume of water consumed was based on a non-linear, temperature-dependent function derived from data presented by Parish and Rhinehart (2008):

$$Liters=33.51213-0.74978*avg\;daily\;temp+0.05806{*avg\;daily\;temp}^{2}.$$ (1)

In this function, average daily temperature (calculated as the average of daily maximum and minimum) determined the total expected liters consumed per day, which was distributed over the total minutes cattle were expected to drink, adjusted for travel time to the lake.

To gain STEC though grazing, cattle had to graze from a patch contaminated with infectious feces. The CFU’s up-taken during a grazing event was proportional to both the amount of CFU’s in the patch, and the amount of grass eaten:

$$STECexposed=Patch\;CFUs*(grass\;units\;consumed|pregraze\;units\;available)*p_{GrazeInfect.}$$ (2)

Populations of STEC shed in feces into water and onto grass were modeled as CFU’s per patch. STEC dynamics in the environment can vary by both environmental factors like temperature and environmental substrate [35–39], and these differences may contribute to environmental transmission dynamics. STEC are known to decay in the environment as a factor of increasing temperature[37,38]. Therefore, we modeled the CFU per patch to decay according to temperature, but at a substrate-dependent rate. In particular, we assumed that STEC decayed faster in water in an agricultural setting [37] than in fecal-pats [38] due to greater competition from other microbial organisms in water in agricultural settings. Decay was modeled in both substrates followed a common Q_10_ function [38,40]:

$$k_{T}=k_{r}{Q_{10}}^{\frac{T-Tr}{10}}$$ (3)

where k_T_ was the bacterial decay rate at given temperature *T* (°C), *k*_*r*_ was the bacterial decay rate at the reference temperature (*Tr*), and Q_10_ was the temperature coefficient that gave the rate of change for each temperature increase of 10°C. Although there is evidence that suggests that a short period of growth can occur in STEC in various substrates following deposition, the accumulated CFUs may be relative insensitive to this initial growth because of the prolonged decay [37,38,41], and thus was ignored here.

The probability of colonization (*p*_*co***l**_) was based on daily accumulated CFU’s and was calculated using a re-arranged form of the Hill-1 dose-response equation presented in [42]:

$$p_{col}=\frac{1}{1+K/CFU}$$ (4)

in which *K* = median infectious dose of the population, and *CFU* was total CFU’s accumulated over the day. Whether or not colonization occurred was determined by drawing a random value from a uniform distribution between 0–1, and assessing whether the value fell below (colonized), or above (not-colonized) p_col_. The source of colonization (direct, water, graze) was assigned based on the category contributing the majority of CFU’s. In addition, the infectious individual responsible for direct transmission or that excreted the cow pat resulting in an indirect colonization was noted during the infectious period of the initially infected individual in order to calculate the basic reproduction number (R_0_), which is the average secondary infections produced by a single infectious individual in an otherwise susceptible population.

### Sensitivity analyses and calibration

To characterize the parameter space of the model and to assess the relative influence of different parameters on dsease dynamics, a two-stage sensitivity analysis was conducted. Both used the count of incident cases at the end of each simulation as an output. First, a local sensitivity analysis was carried out [34] for a subset of parameters (18; 12 deterministic, 6 probabilistic (see Table 1) using either mean values from literature sources (when available) or assumed values as starting values. In this method, the effect of perturbations to each parameter was assessed individually by holding all parameters at their starting values except for the test parameter, which was set to be either greater than or less than its mean or assumed value, with the range tested varying by parameter (see Table 1). In these analyses, the effect of variable shedding rates between cattle was incorporated by randomly sampling a normal distribution with mean equal to the mean *C* value and variable standard deviation. Based on data presented in [43], the effect of variable shedding within cattle was accomplished by assuming that cattle shed the most upon initially becoming infected (i.e., the selected *C* value) and applying an exponential decay function with a variable daily decay rate. All model parameterizations were run under 4 constant temperatures, including cool (20°C) and warm (30°C) temperatures, at *T*_*thr*_ (24°C), and marginally above *T*_*thr*_ (25°C) to differentiate influences on model output due to absolute temperature rather than differences in temperature threshold-dependent Rest behavior. At each selected temperature, each parameter set (37; mean conditions + 36 sets with 1 permuted parameter) was run 100 times for a total of 3700 simulations per temperature. The numbers of incident cases over the simulation period were evaluated for parameter sensitivity, with increases in total cases by at least 100% or decreases by at least 50% indicating a potentially sensitive parameter.

**Table 1:** All parameters used in individual-based simulation model.

Parameter Type	Input/Parameter	Description	Value	Reference	LSA?	LHC?
**Basic Settings**	**Lake position**	Location of lake in pasture	Left side of pasture	Assumed	** **	**
	**Lake size**	Size of lake (hec)	0.41 ha	Assumed	** **	**
	**GWR**	Grass Weed Ratio	4:01	Assumed		**
	**N**_**T**_	Number of Trees	5	Assumed		**
	**R**	Shade Radius (m)	7.6	Assumed		**
	**N**	Cattle Group Size	25	Assumed		**
	**t**	Simulation period (day)	60	Assumed		
	**α**	Grass growth rate (per hour)	1 X 10^−3^	Assumed	*	
	**dailydefavg**	Daily cow pats produced (per cow)	Variable: 11, 15, 17, depending on temperature	[44,45]		
	***avg_mass***	Average mass of a cow-pie (g)	2000	[46]		
**Temperature Sub-models**	***a***	Minimum water temperature (Celsius)	0	[47]		
	***b***	Maximum water temperature (Celsius)	30.4	[47]		
	***c***	Measure of the steepest slope of the function	0.14	[47]		
	***d***	Air temperature at the inflection point (Celsius)	16.5	[47]		
**Bacterial Decay Sub-model**	**k**_**20w**_	Bacterial decay rate in water at 20°C	0.056	[37]		
	**Q**_**10w**_	Coefficient for the change in rate of decay day per day for each 10°C increase of water temperature	1.415	[37]		
	**k**_**20m**_	Bacterial decay rate in manure at 20°C	0.042	[38]		
	**Q**_**10m**_	Coefficient for the change in rate of decay day per day for each 10°C increase of manure temperature	1.48	[38]		
**Animal activities Sub-models**	**T**_**thr**_	Threshold temperature (Celsius)	24	Assumed; similar to [24]		
	***p***_***nearestpatch***_	Probability of selecting the nearest water patch versus a random water patch	0.9 (0.1,0.9)	Assumed	*	
	***p***_***movenewpatch***_	The probability of staying and grazing versus moving to a new patch	0.5 (0.1,0.9)	Assumed	*	
	***p***_***stayanddrink***_	Probability of dominant cow staying in the current water patch to drink	0.9 (0.1,0.9)	Assumed	*	
	***p***_***movetodominant***_	Probability subordinate cow moves towards dominant cow during drinking	0.9 (0.1,0.9)	Assumed	*	
	***p***_***movewhilerest_dom***_	Probability of dominant cow moving while resting	0.1 (0.1,0.9)	Assumed	*	
	***p***_***movewhilerest_sub***_	Probability of subordinate cow moving while resting	0.1 (0.1,0.9)	Assumed	*	
**Epidemiological Sub-models**	**C**	Concentration in feces (CFU/g)	10.36 (0.4, 4)	[48]	*	*
	**ddt**	minimum distance (m) necessary to transfer CFU's directly	0.45 (0.05, 0.5)	Assumed	*	*
	**pln**_**mean**_	Mean of Poisson-lognormal distribution (direct bacterial transfer parameter)	4.72 (2.5, 7.5)	Assumed	*	*
	**pln**_**sd**_	Standard Deviation of Poisson- lognormal distribution	0.5 (0.25,1)	Assumed	*	
	**p**_**Grazenfect**_	Probability of contact with CFU's in a contaminated grass	0.025 (0, 0.1)	Assumed	*	*
	**K**	Dose where 50% of primary susceptible individuals get infected	6.9 x 10^4^ (10^3^, 10^5^)	[16,49,50]	*	*
	**Si**_**mult**_	50% Infectious Dose Multiplier for Secondary Infections	4.722 (1,10)	Assumed	*	*
	***latent***_***phase***_	Exposure period prior to the beginning of shedding (days)	2 (1,3)	[16,17,49]	*	
	**γ**	Recovery time (days)	18.855 (11,30)	[17,49–52]	*	*
	***VarShed***_***within***_	Coefficient of exponential decay of shedding rate per day (starting with 4 CFU per gram of manure)	0 (0,2)	Assumed	*	
	**VarShed**_**between**_	Standard Deviation of normal distribution of mean C of LSA (assumed 4 CFU per gram of manure)	0 (0, 0.1)	Assumed	*	

Parameters either used assumed values, literature sources or were derived from the calibration process. For parameters that were included in calibration and for which a reference is listed, the literature source served as a starting value. If multiple sources are listed, either the value is an average (*latent*_*phase*_), or multiple values were used in establishing the starting value and/or range for the calibration (*K*, *γ*). LSA = Local sensitivity analysis; LHC = Latin Hypercube Cube. For LHC

* = used to calibrate final model

** = structural feature assessed using the calibrated model.

From this local analysis, 7 parameters identified as “sensitive” (shown later in results section) were included in a global Latin Hypercube Sampling (LHS)-based sensitivity analysis. Parameters were randomly sampled in each 0.1% of their parameter space, resulting in 1000 unique parameter sets. Model runs were completed for each parameter set under the same set of 4 constant temperatures as previous (20°C, 24°C, 25°C, and 30°C). Then, partial rank correlation coefficients (PRCC) and corresponding 95% confidence intervals (via 100 bootstrapped samples) were computed for each parameter in each temperature set [53] using the package sensitivity in *R* [54].

To calibrate the model, we used a method similar to the “best fit” method suggested by Railsback and Grimm (2012) [34], in which model outputs were calibrated against aggregate estimations of winter (95% CI: 1.50–9.49%) and summer (95% C: 7.98–16.25%) STEC prevalence reported for pastured beef cattle in the United States [55]. This proceeded by selecting the subset of LHS model runs (at 20°C) with a prevalence over the 60 day run that fell within the range (1–2 new colonizations) of reported winter prevalence. Because the model population was small (25), and all model runs resulted in at least 1 infection (the starting individual), prevalence at the end of the run was assessed as: the number of incident cases / 24. The median of these parameter values was then used as the parameter set of a series of simulations run at 20°C, but with incremental changes (0.01–0.04 in increments of 0.005, 100 simulations apiece) to the value of *P*_*GrazeInfect*_. This parameter was shown by PRCC to have a relatively strong individual influence on incidence at 20°C, to have relatively low influence at 30°C, and has considerable uncertainty, making it a desirable candidate for calibration [34]. From these simulations, a value of *P*_*GrazeInfect*_ was selected that resulted in the average prevalence (of the *P*_*grazeinfect*_ level) at the end of the model run falling in the calibration range, and near to the mean value presented in Ekong et al. (2015) [55].

Next, model simulations were run at 30°C using the resulting parameter set, but in which the value of the distance threshold at which direct transmission was possible (*ddt*) was incrementally adjusted (0.2–0.5, in increments of 0.05, 100 simulations apiece). Similar to *P*_*GrazeInfect*_, *ddt* was chosen as a variable to manually calibrate because PRCC analysis showed that it was relatively influential at 30°C, relatively non-influential at 20°C, and to have high uncertainty. As before, a value of *ddt* was selected from model runs that resulted in the average prevalence (of the *ddt* level) at the end of the model run falling in the calibration range, and similar to the mean value presented in Ekong et al. (2015)[55] for summer STEC prevalence in the United States. Lastly, 1000 simulations were run using the final calibrated set at both 20°C and 30°C to verify that simulation outputs fell into the expected ranges.

Lastly, to gauge the influence of several simplifying assumptions made in the calibrated model, we conducted a second LHC-type sensitivity analysis in which aspects of the physical environmental structure and cattle population were varied. These included the number of trees, the radius of the shade patch, the size, arrangement, and location of the water source, the proportion of weeds to grass, and the number of cattle per simulation. See S2 Text for a description of this analysis and detailed results.

All parameter values used in model simulation, as well as ranges tested during sensitivity analyses used for calibrated are shown in Table 1. All analyses were conducted using program *R* with various packages [56]. Simulations were prepared using the RNetLogo package [57], and run on either a desktop computer or the North Carolina State University High Performance Computing (NCSU-HPC) cluster. The model can be found in S1 Model and its associated temperature files and an example R script to run it can be found in S1 Folder.

### Factorial analysis

We evaluated our hypotheses regarding how temperature influences STEC dynamics using a fully factorial design that compared simulation results from scenarios assuming three different seasonal temperature conditions, including spring (beginning cool with a warmer end), summer (warm throughout), and fall (beginning warm with a cooler end) conditions. Within each temperature condition, assumptions of temperature-dependent and temperature-independent cattle behaviors, including daily resting behavior conditions (3 states) and drinking behaviors conditions (2 states) were systematically varied for a total of 6 factorial combinations. Resting behavior states included 1) always exhibiting ≤ T_thr_ Rest/Graze behavior (“Rest Cool condition”), 2) always > T_thr_ behavior (“Rest Warm condition”), and 3) exhibiting temperature dependent behavior (“Rest Dep condition”). Drinking behavior states included 1) temperature-dependent (“Temp Dep condition”) or 2) constant (“Temp Indep condition”), assuming drinking rates at 20°C. For each factorial combination, historic temperature (see S1 Folder) in each of 10 years (2002–2011) were used to run 100 simulations for two spring months (April and May), two summer months (June and July), and two fall months (October and November) of that year, with a total of 18000 simulations, each of 60 days apiece. During this 10-year period, summer temperatures were relatively stable, with an overall mean of 25.9 ± 1.2°C, and mean highs and lows during summer were 31.3 ± 1.5°C and 20.4 ± 0.9°C, respectively. Spring temperatures increased over the course of the 60-day model run, with a mean temperature of 17.9± 0.9°C, an average high of 23.9± 1.01°C, and an average low of 11.8 ± 0.9°C. Fall temperatures decreased over the course of the 60 day model run, with a mean temperature of 12.8± 1.1°C, an average high of 18.8± 1.2°C and an average low of 6.7± 1.3°C.

Model outputs from each simulation included the count of incident cases over the duration of the simulation (and prevalence over the period) and on a daily basis the relative proportion of incident cases originating from each transmission pathway of colonization (i.e. direct, indirect water, indirect graze), and *R*_*0*_. Because colonization pathways are generally unknown in real-world systems, non-source specific incident cases over the duration of the simulation were the primary output of interest for analysis. However, the explicit accounting of colonization pathways in the model enabled us to relate general patterns of incident cases with the pathways driving those patterns.

Initial exploration showed a high proportion of simulations resulted in zero new colonizations, depending on the model conditions. To understand drivers of both epidemics occurring and their extent, we modeled incident cases in two stages using an approach similar to that of a zero-altered generalized linear model (ZAGLM) (otherwise known as a hurdle model [58]) with the glmmADMB package in *R* [59,60]. In the first of this two-part approach, the probability of 0 new colonizations was modeled with a binomial distribution. In the second part, incident cases > 0 were modeled with a zero-truncated negative binomial distribution. This approach acknowledged that zero new colonizations could emerge as a result of both underlying system stochasticity and model conditions (with particular probability), but unlike observed systems in which false zeros occurred and in which zero-inflated approach would be appropriate [61], all colonizations were captured by the model. For both the binomial and count models, the β and ϒ vectors were the same, with the linear predictor structure:

$$\eta (Y_{i})=\mu +R+D+(R*T)+(D*T)+ᴇ$$ (5)

In this equation, the link function *η(Yi)* is either the logit link ($\frac{1}{1+e^{-\pi }}$) of the binomial distribution (giving the probability of 0 colonizations in year *i*), or the log link (ln *π*) of the negative binomial distribution (giving the actual count of colonized individuals in year *i)*; μ was the mean of the binomial or zero-truncated negative binomial distribution; R, D, and T were the fixed effects of rest behavior, drinking behavior and temperature, respectively; R*T and D*T were interactive terms between drinking and rest terms and temperature, respectively, and *E* represented independently distributed error. A comparison to a similar mixed model structure that included year as a random intercept term found the two model structures did not significantly differ in their fit (according to a Likelihood Ratio Test, data not shown), and that the non-mixed models had a lower AIC value. Therefore, the non-mixed model was selected for analysis here. Other model outputs, including incident cases from direct and indirect pathways, and *R*_*0*_ were summarized in relation to the incidence model. Lastly, an explicit comparison was made of model outputs across seasons using the temperature-dependent parameterizations of both resting and drinking behavior.

## Results

### Sensitivity analysis

**Fig 3:**
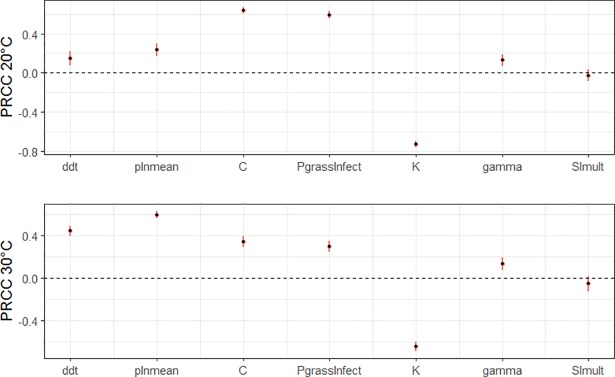
Influence of parameters depends upon whether the temperature is ≤ or > the Graze/Rest behavior threshold (24°C). 95% CIs of partial rank correlation coefficients (PRCC) of simulations parameterized with 1000 unique parameter sets of 7 variables derived from a Latin Hypercube-based approach. Simulations run at constant 20°C, 24°C, 25°C, and 30°C to differentiate temperature-threshold versus continuous temperature effects. PRCCs at 24°C and 25°C were found to be similar to PRCC’s at 20°C and 30°C respectively, and are not shown here. Please see S2 Text for PRCC metrics at all temperatures. Parameters include: *ddt* = distance of direct contact; *pln*_*mean*_ = mean of the Poisson-lognormal distribution (sampled for direct transmissions), *C* = STEC concentration in cow pats; *P*_*grassinfect*_ = proportion of CFU’s up taken per contaminated grass unit, per grass patch when grazing; *K* = median population dose of STEC expected to result in colonization; **γ** = recovery time (days); and *SI*_*mult*_ = amount *K* multiplied by in the case of secondary colonizations.

The LSA procedure identified 7 particularly sensitive variables for inclusion in the LHS, including the distance threshold at which direct transmission was possible (*ddt*), the probability of ingesting STEC via grazing a contaminated patch (*p*_*GrazeInfect*_), the recovery time following colonization (**γ**), the mean of the Poisson-lognormal distribution sampled to determine the quantity of STEC transferred during direct transmission events (*pln*_*mean*_), the concentration of STEC in a new fecal-pat (*C*), the concentration of *STEC* expected to infect 50% of exposed individuals (*K*), and the factor (*SI*_*mult*_) by which *K* was multiplied to simulate partial immunity for previously infected individuals to secondary colonizations. Boot-strapped estimates of PRCC’s at each temperature set showed very low bias (<0.01) for each variable (indicating high stability of estimates), and the 95% CI’s of all variables except *SI*_*mult*_ did not include 0, indicating statistically significant correlations. PRCC estimates demonstrated that the sensitivity of incident cases to changes in parameters generally depended on the temperature (Fig 3). At the cooler temperature sets (20°C, 24°C), *C* and *p*_*GrazeInfect*_ were strongly positively correlated with counts of incident cases while only weakly correlated at warmer temperatures (25°C, 30°C). Inversely, *ddt* and *pln*_*mean*_ were strongly positively correlated with counts of incident cases at warmer temperatures while only weakly correlated at cooler temperatures. Lastly, sensitivity to some parameters was independent of temperature, including the strongly negatively correlated *K*, and the weakly correlated parameters *SI*_*mult*_ and **γ**. Differences in PRCC between simulations at 20° and 24° C and between simulations at 25°C and 30°C simulations were minimal, indicating that sensitivity to parameters was more strongly influenced by model behavior determined by the 24°C temperature-threshold than continuous changes in temperature.

The secondary LHS sensitivity analysis (S2 Text) found grass-to-weed ratio to have a significant, but weak positive correlations (PRCC ≤ |0.3|) with incident case counts at both 20° and 30°C. Meanwhile, the total number of cattle in the simulation was moderately positively correlated with incident case count (PRCC = 0.44) at 30°C, and more weakly correlated (PRCC = 0.34) at 20°C. In addition, although the size of the lake did not have significant influence on incident cases, there were consistently more incident cases if the lake was positioned at the corners of the rectangular pasture than if the lake was positioned at the sides or the center.

### Factorial simulations

The binomial model found that the probability of zero new transmissions relative to baseline conditions (spring temperatures, Rest Cool, Drink Indep) was driven by significant interactions between temperature and Rest behavior condition (Table 2). Together, the interactions show that the probability of zero transmission is reduced (relative to spring temperatures) when the Rest Warm or Rest Dep conditions occurred with summer temperatures. In contrast, increasing temperature alone increased the probability of zero new colonizations, likely due to higher bacterial degradation rates at higher temperatures. Drinking behavior had no clear impact on the probability of zero new transmissions.

**Table 2:** Temperature and Rest/Graze behavior interact to determine the likelihood and extent of epidemic.

Model Type	Effect Type	Variable	Estimate	Standard Error	Z-score	P-value
Binomial-Model	Main Effects	Intercept	-0.27331	0.05244	-5.212	<0.001
		Rest Warm	-0.44444	0.06585	-6.749	<0.001
		Rest Dep	-0.06995	0.06415	-1.09	0.276
		Drink Dep	-0.05395	0.05329	-1.012	0.311
		Summer Temperature	0.72139	0.07446	9.689	<0.001
		Fall Temperature	-0.06009	0.07429	-0.809	0.419
	Interaction Effects	Rest Warm * Summer Temp	-0.40743	0.09234	-4.412	<0.001
		Rest Dep * Summer Temp	-0.77769	0.09113	-8.534	<0.001
		Rest Warm * Fall Temp	0.06333	0.093	0.681	0.496
		Rest Dep * Fall Temp	-0.09902	0.09107	-1.087	0.277
		Drink Dep * Summer Temp	-0.0762	0.07511	-1.015	0.31
		Drink Dep * Fall Temp	0.07109	0.07548	0.942	0.346
	N = 18000					
	Null Deviance: 24308 on 17999 df					
	Residual Deviance: 23887 on 17988 df					
Model Type	Effect Type	Variable	Estimate	Standard Error	Z-score	P-value
Count Model	Main effects	Intercept	0.7572	0.0308	24.55	<0.001
		Rest Warm	0.8058	0.0329	24.51	<0.001
		Rest Dep	0.2716	0.0371	7.32	<0.001
		Drink Dep	-0.0426	0.0245	-1.74	0.0815
		Summer Temperature	-0.3841	0.0541	-7.1	<0.001
		Fall Temperature	0.0482	0.0428	1.13	0.2605
	Interaction Effects	Rest Warm * Summer Temp	0.1793	0.0568	3.16	0.002
		Rest Dep * Summer Temp	0.6975	0.0594	11.74	<0.001
		Rest Warm * Fall Temp	-0.0261	0.0458	-0.57	0.5683
		Rest Dep * Fall Temp	-0.1363	0.0521	-2.61	0.009
		Drink Dep * Summer Temp	0.0977	0.0354	2.76	0.006
		Drink Dep * Fall Temp	0.0165	0.0345	0.48	0.633
	N = 10699					
	Negative Binomial Dispersion Parameter: 1.58					

Results of binomial model of zero-new colonizations occurring (all data, dichotomized) and negative binomial (count) model of incident cases (>0) produced by ABM model simulations. All simulations used daily maximum and minimum temperatures data collected from 2002–2011 from weather station (S1 Text and S1 Folder). Spring Temp = April-May; Summer Temp = July-August; Fall Temp = October-November. Rest Cool condition = cattle always rest in the field and spend more time grazing; Rest Warm condition = cattle always rest under trees as a group and spend less time grazing; Rest Dep condition = cattle rest/graze depending on temperature.

**Fig 4:**
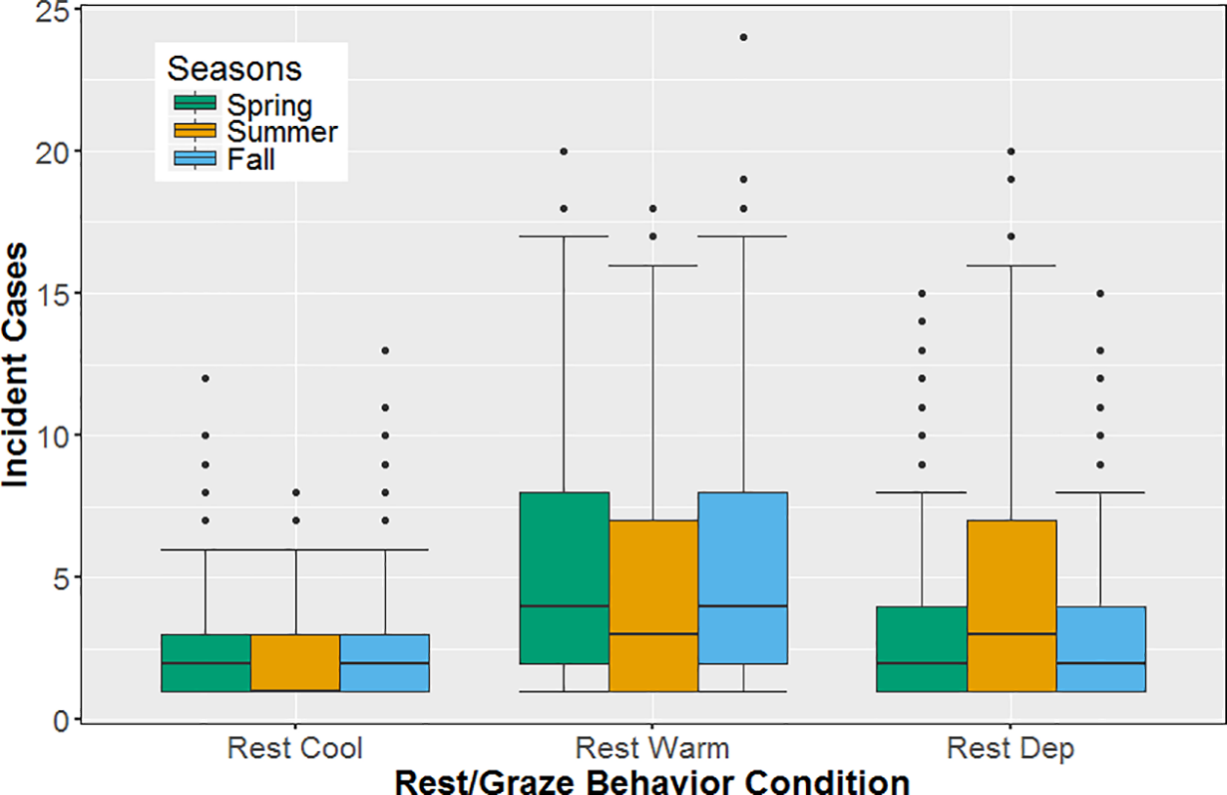
Grazing less and resting under trees increased the extent of epidemics. Counts of incident cases by Rest/Graze condition; simulations resulting in zero colonizations excluded. Rest Cool condition = cattle always rest in the field and spend more time grazing; Rest Warm condition = cattle always rest under trees as a group and spend less time grazing; Rest Dep condition = cattle rest/graze depending on temperature.

Results of the incident case model (using non-zero counts) were similar to the binomial model, with significant interactions between rest behavior condition and temperature defining the number of non-zero incident cases over the model run (Table 2). In general, interactions with rest behavior condition had the largest impact, with the Rest Warm and Rest Dep conditions resulting in higher average incident cases than the Rest Cool condition (Fig 4). Within the Rest Cool and Rest Warm conditions, the count of incident cases were similar across seasons, although higher bacterial degradation rates resulted in the lowest incidences with summer temperatures under both Rest Cool and Rest Warm conditions. With the Rest Dep condition, a large positive interaction with temperature resulted in the highest average count of incident cases during the summer. This was due to warmer temperatures leading to Rest Warm behavior (i.e., resting under trees) occurring on most days in summer simulations. Average counts of incident cases were also higher with spring temperatures than fall temperatures under the Rest Dep condition, likely due to warmer overall temperatures during the spring than the fall (particularly during the latter half of it), and therefore more days with Rest Warm behavior than conditional Rest Cool behavior. In contrast to the binomial model, a significant interaction also occurred in which the count of incident cases as higher with summer temperatures under a temperature-dependent drinking condition.

#### Differential transmission pathways

**Fig 5:**
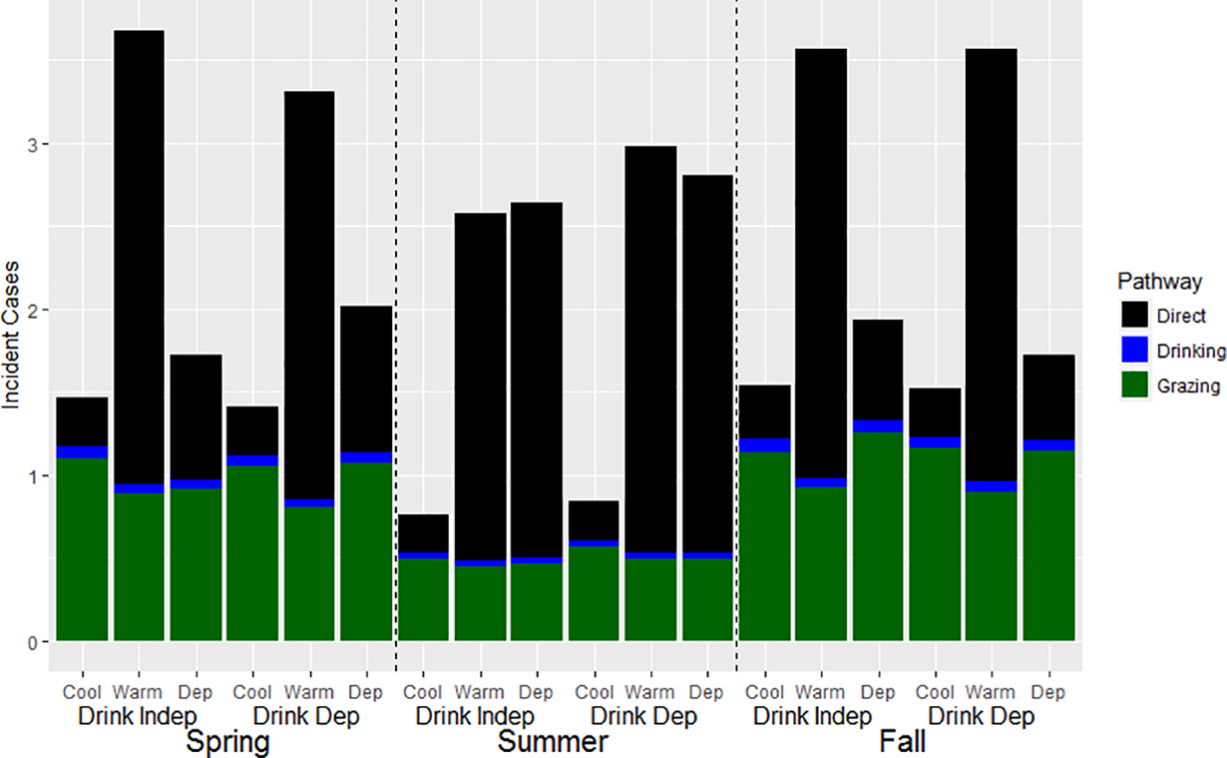
Transmission pathways were dependent upon seasonal temperature and Rest/Graze behavior. Proportion of total average counts of incident cases accounted for by each pathway for each factorial combination of Rest/Graze and Drinking condition. Cool condition = cattle always rest in the field and spend more time grazing; Warm condition = cattle always rest under trees as a group and spend less time grazing; Dep condition = cattle rest/graze depending on temperature. Drink condition = Dep if drinking condition was dependent on temperature; Drink condition = Indep if drinking rate was assumed static (at 20°C rate).

Considering contributions to counts of incident cases from different transmission pathways showed that the distribution of colonizations between pathways depended largely on rest condition and seasonal temperature (Fig 5). When considering the proportion of total transmission occurring within a simulation, transmission under Rest Warm conditions largely occurred through direct transmission, and the grazing pathway accounted for the majority of new colonizations for seasons under the Rest Cool condition. In contrast, the majority pathway under the Rest Dep condition depended on season, with the majority of new colonizations during the spring and fall temperatures transmitted through grazing, and the majority of transmission during the summer occurring through direct contact. Overall, transmission via water was generally minimal, and drink behavior condition had little discernable impact on the distribution of colonizations between the transmission pathways. For all seasonal temperatures, secondary transmission did not appreciably contribute to the count of incident cases, accounting for an average low of 0.93% (Spring) to an average high of 1.1% (Fall) of incident cases overall.

#### Basic reproduction number

**Fig 6:**
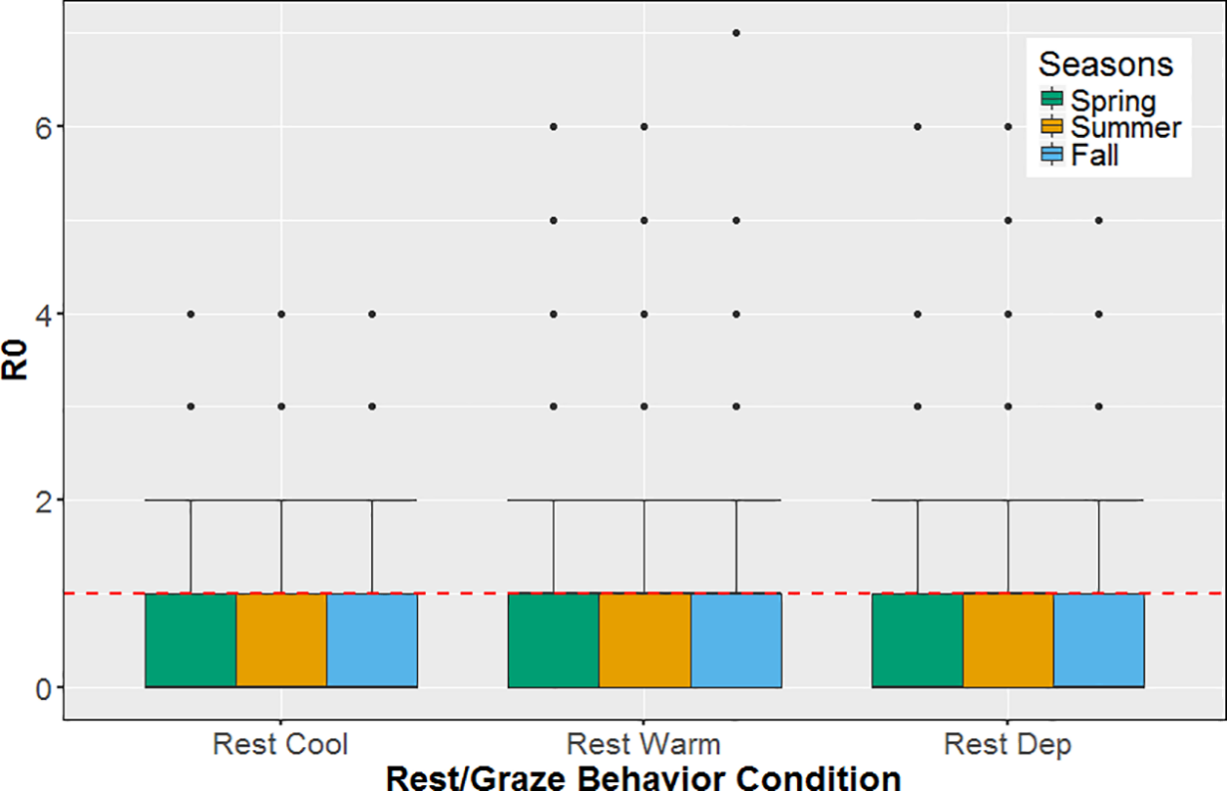
R_0_ was dependent on interaction of seasonal temperature and Rest/Graze condition. R_0_ by Rest/Graze condition. Median is black line in each bar. Epidemic threshold (R_0_ = 1) is the dotted red line. Rest Cool condition = cattle always rest in the field and spend more time grazing; Rest Warm condition = cattle always rest under trees as a group and spend less time grazing; Rest Dep condition = cattle rest/graze depending on temperature.

The *R*_*0*_ varied significantly with temperature and Rest/Graze conditions, largely mirroring the pattern found for incident cases (Fig 6). Under Rest Cool conditions the distribution of R_0_ was similar cross seasons. Of note here was that the average *R*_*0*_ under Rest Cool conditions across seasons (0.43) is << 1, reflecting the high proportion of zero new transmissions under this condition. In contrast, while average *R*_*0*_ with summer temperatures was similar under both Rest Warm and Red Dep conditions (0.82 ± 0.1 SE), average R_0_ values were higher with cooler seasonal temperatures under Rest Warm conditions (Fall: 0.86 ± 0.1 SE; Spring: 0.95 ± 0.1 SE), and lower with cooler seasonal temperature under Rest Dep conditions (Fall: 0.55 ± 0.08 SE; Spring: 0.58 ± 0.08 SE). This reflected the influence of bacterial degradation in the environment on transmission, with higher rates of decay occurring during the warmer months.

#### Temperature-dependent conditions

Of the total set of factorial model runs, those operating under model conditions of Rest Dep and Drink Dependent reflected comparisons of fully temperature-dependent parameterizations of the model during different seasons; that is, models operating under conditions most representative of real-world conditions. When considered in terms of prevalence (considered here as the count of incident cases / 24), the 95% CI for average prevalence using summer temperatures (0.09–0.15) fell within the empirical 95% CI of summer STEC used as a validation range (7.98–16.25) [55]. Meanwhile, the 95% CI’s for average prevalence during the spring (0.06–0.1) and fall (0.055–0.089) largely overlapped with empirical 95% CI of winter STEC prevalence used as validation range (0.015–0.0949) [55]. That the means of the intervals of the spring (0.084) and fall (0.072) simulations were higher than the validation range mean (0.048) is likely a result of temperatures > *T*_*th*r_ during the end of the spring and beginning of the fall that drove colonizations higher than in the calibration sets, all run at a constant 20° C.

## Discussion

Higher STEC prevalence in environmental substrates, feces, and beef carcasses, as well as an increase in shedding of STEC by cattle during warmer seasons of the year has been commonly reported [8,11]. For example, Barkocy-Gallagher et al. (2003) [62] reported mean STEC prevalence of 12.9%, 6.8%, and 3.9% during Summer, Fall, and Spring seasons in samples taken beef carcasses in a beef processing facility. Van Donkersgoed et al. (1999) [63] reported higher prevalence of STEC in fecal samples from cattle at slaughter during the summer months than cooler months, and a large-scale review (used for calibration purposes here) reported mean values of winter and summer prevalence in US pasture-range beef cattle of 4.84% and 11.82%, respectively [55]. Various mechanisms have been postulated to explain this phenomenon (Fig 1), and two hypotheses were comparatively evaluated here, including increased drinking with higher temperature (and thus more water-based transmission) and more frequent aggregation under shade trees as temperatures increases, promoting more direct transmission.

The results of the factorial analysis of model simulation outputs found that temperature-induced changes in rest behavior most strongly drove overall patterns of new colonizations. Counts of incident cases were significantly higher when either 1) cattle always rested under shade trees versus resting in place and grazing an extra hour, or 2) when rest-behavior was temperature dependent and temperatures were more frequently above the temperature threshold (*T*_*thr*_*)* causing cattle to rest under trees more often. The average temperature under summer conditions was above *T*_*thr*_ (25.6°C) while the average temperatures during spring (17.9°C) and fall (12.8°C) were below it, resulting in the highest average count of incident cases with summer temperatures. Further, direct transmission was the dominant transmission pathway during these higher incident case situations, where it accounted for >74% of new colonizations for all Rest Warm factorial combinations, and for > 79% of new colonizations for Rest Dep conditions during the summer (Fig 5). Meanwhile, graze-based transmission was the primary pathway in all other situations. In addition, the probability of no new colonizations occurring was significantly negatively impacted by the Rest Dep/Rest Warm conditions, particularly under summer conditions, meaning that temperature-driven spatial aggregation both significantly increased the probability of an epidemic occurring at all, as well as determining its extent.

In contrast, higher drinking rates had a more limited effect on STEC incidence in which an interaction with summer temperatures resulted in slightly higher counts of incident cases through a drinking-pathway during this season (Fig 5). However, the proportion of incident cases due to water was low small relative to other pathways, accounting for a maximum of 5% of colonizations in any factorial combination (Fall, Rest Dep Conditions). This contrasts with previous research showing that drinking water is a plausible transmission pathway for STEC [11]. The water resource in the model system was a single 1-acre lake of uniform 0.5 m depth instead of water troughs, as in previous work. Thus, the dilution of concentration and dispersion of fecal-pats due to volume within water patches likely contributed towards the reduced contribution of water-based transmission. However, partially compensating for this was the assumption that STEC concentration within a particular water patch was homogeneous throughout the water column, and was directly proportional to the concentration in the deposited fecal-pat. In real systems, the concentration of STEC cells would be partitioned between aquatic and sediment phases due to adsorption [64], and therefore the availability of STEC for ingestion may be limited. On the other hand, the relatively high daily decay rate at 20°C (0.388) compared to the low rate (0.042) used for decay in manure may have underestimated the persistence of STEC in water. On the balance, however, STEC in water was likely more available for uptake than in a real aquatic system, increasing the likelihood of transmission. Thus, of the two hypotheses, temperature-driven spatial aggregation that promotes a greater frequency of direct contact between individuals provides the more plausible mechanism to explain seasonality in STEC prevalence in grazing systems, as least when water resources are similarly structured.

Increased direct transmission through temperature-mediated spatial aggregation is a plausible explanation for seasonal patterns in STEC transmission for several reasons. First, increasing animal density is well understood to be positively associated with the transmission of infectious disease [65], and there are previous reported instances in which higher STEC prevalence in cattle may have been due to increased aggregation in the absence of warmer temperatures. In particular, cattle may have a higher risk of shedding STEC when housed than pastured [66,67], even during cooler months [68]. Secondly, climate varies widely in space, and the temperatures (collected near Knoxville, TN), schedule and spatial structure of the model assumed here are not representative of conditions in many other locations. Therefore, it is not unexpected that there are reported instances in which the pattern of STEC prevalence did not vary strongly with season [69], or was not clearly associated with increasing temperature [70,71]. In one of these cases, however, the prevalence of STEC in feed lot cattle was found to increase with time after cattle arrived in the yard [71]. Thus, changes in spatial aggregation patterns with temperature, rather than changes in temperature alone, may be a reasonable underlying mechanism to explain seasonal STEC prevalence where it occurs. Less clear, however, is whether direct transmission would be the dominant pathway, as suggested by the model.

Direct transmission is generally thought to occur via a fecal-oral route, either from social interactions (e.g. grooming activities) which result in transfer of STEC via direct ingestion [17], or from incidental contacts due to proximity that can result in the transfer of feces between hides [17]. The transmission of STEC via aerosols between cattle in close proximity has also been suggested [16]. In the model, these forms of contact are not differentiated, with a quantity of CFU’s per contact drawn from a distribution whenever a contact occurred due to the breach of the distance threshold. The Poisson-lognormal distribution sampled to simulate the transference of STEC during a contact is integer-valued and over-dispersed [72], meaning that large numbers of STEC are rarely transferred when direct contacts occur. Because they are directed and may last several minutes [73], social interactions such as allo-grooming may have the potential to transfer enteric pathogens more efficiently than incidental contacts. However, social interactions occur non-randomly and often occur hierarchically, with less dominant individuals being groomed by more dominant ones [74]. The Poisson-lognormal distribution used approximates the condition that most contacts between cattle are incidental (transferring smaller quantities of STEC), while some are social (transferring larger quantities). Because the nature and context of cattle contacts were not explicitly modeled here, there is uncertainty in understanding how aggregation, beyond the simple proximity rules used in this model, may influence direct transmission. Additional work explicitly incorporating more complex social structure into direct contact behavior could be helpful in reducing this uncertainty.

**Fig 7:**
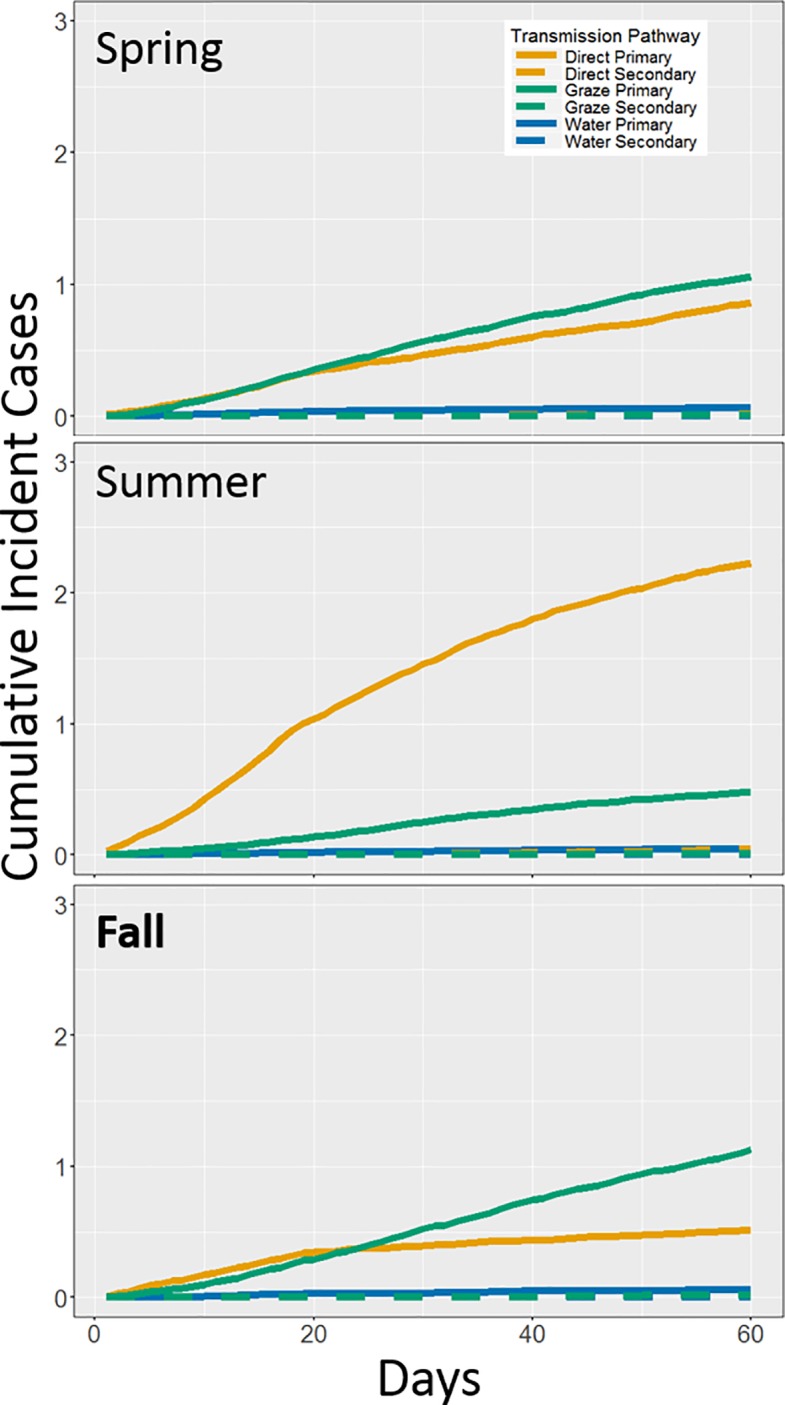
Seasonal temperatures drove distribution of transmission pathways due to Rest/Graze behavior. Cumulative counts of incident cases for direct, graze-based, and water-based transmission pathways over the duration of simulations (60 days) assuming full temperature dependent conditions. Primary transmission is indicated by solid lines while dashed lines indicate secondary transmission, clearly showing that secondary transmission uncommonly occurred over the relatively short time period of the simulation.

Indirect transmission through the ingestion of contaminated grass emerged as the most important pathway during spring and fall under the Rest Dep condition, and under the Rest Cool condition. That the graze-based pathway was more important than the water pathway may have partially been because even though STEC decayed with rising temperature in both substrates, STEC decayed faster decay in agricultural water [37] than in fecal-pats [38] due to greater competition from microbial organisms in the former (Table 1). It should be noted that this may not be the case in more pristine water like lakes in non-agricultural settings [37]. When considering model simulations under temperature-dependent conditions, the grazing pathway in all seasons tended to develop at a much slower, approximately linear rate compared to the approximately logistic growth of the direct pathway in the summer (Fig 7). Thus, the grazing pathway may contribute towards maintaining enteric pathogens within a population in an endemic state, particularly at cool temperatures which promote a slower decay of STEC populations in the environment [11]. The greater proportion of graze-based transmission occurring during fall simulations than spring simulations under the Rest Dep condition (Fig 5) was a result of fall temperatures that were cooler on average than spring, resulting in more days of additional grazing. In contrast, higher counts of incident cases during the spring than the fall was the result of more frequent warm weather, resulting in more aggregation and direct-transmission. Overall, these results suggest that higher exposure during grazing can result in a greater proportion of graze-based colonizations even though the chances of ingesting STEC via grazing are low, and that contributions from indirect pathways may maintain low-levels of colonization during cooler weather.

### Model limitations and considerations

While factorial simulations from our model suggest that higher prevalence of STEC in cattle during the warmer months may be due to more aggregation that drives direct transmission, the global sensitivity analyses suggested that the parameter space exists in which environmental transmission may drive counts of incident cases to be similar to or higher during cooler months. The PRCC’s calculated as part of the global sensitivity analysis at each static temperature (20, 24, 25, 30°C) showed clear temperature-driven patterns in sensitivity reflecting differences in the importance of transmission pathways between cool (20 and 24°C) and warm (25 and 30°C) model conditions due to rest behavior below and above the *T*_thr_ (Fig 3). When temperatures were < *T*_thr_, the parameters with the strongest correlations were those influencing transmission via a graze-based pathway (the grass infection factor (*p*_*grassinfect*_) and the starting concentration of STEC in a fecal-pat (*C*)). When temperatures were > *T*_thr_, the strongest positive correlations were those involved in direct transmission, including the direct distance threshold (*ddt*) and the mean of the Poisson-lognormal distribution used to determine the CFU’s of STEC transferred between cattle during direct transmission events **(***p*_lnmean_). So, factors increasing the probability of transmission from a grazing-route could potentially result in higher counts of incident cases during cooler weather than warmer weather. Of the two variables, increasing concentrations of STEC in fecal-pats (*C*) is more likely to occur in natural grazing systems, as cattle are known to avoid eating grass contaminated with feces. Although the quantity of STEC resulting from the calibration process (10.36 g/CFU), was well within the range of values reported (most probable number) of STEC gram^-1^ of cow manure in a study by Fegan et al. (2004) [48], it was far below the maximum value reported in that study (4.3 x 10^2^). It also below the 10^3^ CFU gram^-1^ suggested as the threshold for an individual to be classified as a “super-shedder” [75]. Indeed, an examination of our LHC simulation outputs in which values of *C* > = 10.36 CFU’s gram^-1^ showed mean incident cases to be 20 ± 0.62 SE and 18.66 ± 0.45 for simulations with temperatures of 20°C and 30°C, respectively. In this “high *C*” subset, grazing-based transmission made up 83% of new colonizations at 20°C and 61% of new colonizations at 30°C, respectively. Many animals colonized with enteric pathogens shed heterogeneously over the course of their infectious periods, and evidence suggests that super-shedders may largely be animals sampled near the high-shedding points of their infectious periods [76]. Although variable shedding between or within individuals was not found to have a large effect in on incident case count in our local sensitivity analysis, we did not directly include the presence of “super-shedding” individuals as a parameter or as a hypothesis to be explored. This effect may be further heightened if potential growth of STEC after deposition is considered [37,38,41]. Therefore, the current model may not adequately capture the role of *C* in STEC transmission dynamics.

More frequent graze-based transmission may also occur due to the structure of the model environment and cattle density. Although mostly weak correlations were found between counts of incident cases with environmental structures of the model (S2 Text), positioning the lake into the corners of the property versus the sides or center resulted in more graze-based cases. This appears to occur because when the water source is in a corner of the rectangular model area, cattle tended to concentrate at one end of the property while grazing and were exposed more often to contaminated graze. Although an artifact of model structure here, increasing distance between water and grazing forage has been shown to reduce the use of available forage in grazing cattle [77], and to increase the unequal distribution of manure in pasture systems [78].

Although the model predicts that there are potential pathways for high graze-based transmission, there is currently limited evidence of food-based infections/colonizations [11]. There is also limited evidence that super-shedders contribute highly to increased risk from environmental pathways [75]. In addition, since cattle are known to avoid contaminated graze (accounted for implicitly here by making the CFU of STEC taken up by grazing very low compared to the amount in the plot), it is possible that the model overestimates the potential of graze-based colonization. If so, it would help explain why the 95% CI of prevalence values under temperature dependent conditions during Spring and Fall temperatures fell on the higher end of the calibration range. However, the influence of water location versus graze availability on graze-based exposure worth may be worth exploring in future work, particularly in situations where graze quality widely varies [77].

Lastly, the model assumed that cattle behaved according to simple rules, and that all individuals were of indeterminate adult age. Although sensitivity analyses suggested that increasing cattle density could increase direct transmission at high temperatures due to denser clustering around shade-trees during rest, this assumes that all cattle would always rest under the same tree, and that inter-cattle distances between individuals would not be maintained as cattle density increases. However, maintenance of a minimum personal space is an important aspect of cattle social behavior [74], and cattle may maintain larger distances as herd size increases to reduce aggression [74,79]. So, it is likely that cattle that do not fit under the shade of a tree due to lack of space would find another tree, and that some minimum inter-cattle distance may be maintained during resting. Thus, the influence of increasing density on direct transmission may be overestimated. In contrast, the more moderate effect of increasing cattle density on graze-based transmission may be more mechanistically plausible (i.e., more cattle produce more manure), but because actual stocking rates are determined by pasture yields [80], and transmission was found to decrease as the grass to weed ratio decreased, this relationship may not be practically relevant to lower producing pastures. Finally, the model was calibrated using a meta-analysis of adult beef cattle prevalence data that listed relatively low average values for summer (11.83) and winter (4.84) prevalence in the United States [55]. However, the prevalence of cattle may be significantly influenced by individual factors such as age [81], with the highest expected STEC prevalence during the first year of life [82], and parity status influencing STEC colonization thereafter [81]. Thus, the current model structure and calibration may not adequately capture the temporal dynamics of shedding patterns for juveniles, or different age and parity classes of adult female cattle.

Despite its limitations, the current model structure is quite flexible, and additional ecological, behavioral or biological aspects of agents or the environment can be readily incorporated in order investigate additional hypotheses, or to more closely model particular conditions. Additionally, distinguishing between direct and indirect transmission pathways using empirical data is difficult, particularly if the time-scales of epidemiological dynamics and pathogen dynamics in the environment are convergent [83]. In simulation model-based approaches like the one used here, uncertainty associated with transmission sources within simulations can be eliminated or greatly reduced, making it a useful tool for inferring the role of different pathways in epidemiological dynamics.

## Conclusions

Model simulations suggest that seasonal patterns of higher STEC prevalence during warmer months in some grazing systems may be driven by temperature-mediated aggregation that promotes direct-transmission of STEC between individuals. In the model, this hypothesis is contingent on the presence of shade-providing structures, such as trees, under which cattle aggregate for temperature relief, a centrally located water source, and on the assumptions that cattle follow a rigid social structure in which individuals in the herd follow a dominant individual to resting locations. Therefore, determining ways to reduce rates of close contact between cattle under shade or while being housed could be beneficial to reducing rates of STEC transmission.

## Supporting information

S1 Text Overview, description, and details of model. (DOCX)

S2 Text Sensitivity analyses. (DOCX)

S1 Model NetLogo grazing model. (NLOGO)

S1 Folder Temperature files and R script. (ZIP)
