# Supplementary material for: Investigating behavioral drivers of seasonal Shiga-Toxigenic Escherichia Coli (STEC) patterns in grazing cattle using an agent-based model
Source: PLoS One. 2018 Oct 10;13(10):e0205418. doi: 10.1371/journal.pone.0205418 (PMC6179278; doi:10.1371/journal.pone.0205418)
Supplement: S1 Text — (DOCX) [file pone.0205418.s001.docx]

#### Supplementary Materials S1: Model Overview, Design Concepts, and Details

#### 1. Model Overview

This is a spatially explicit agent-based model (ABM) constructed to simulate transmission of STEC among grazing cattle. The model was written and executed in NetLogo [1] (versions 5.3.1 and 6.0.4), an open-source agent-based modeling software.

#### 1.1 Purpose

The purpose of this model was to quantify how temperature fluctuation caused changes in STEC incidence among grazing cattle by influencing diurnal behavior patterns, including time spent engaged in different activities (grazing, resting, drinking, sleeping), and volume of water consumption.

#### 1.2. Entities, State Variables, and Scales

The simulation model consisted of a 101 x 221 grid system resulting in 22,321 unit square patches. The grid system represented a 20-acre typical pasture where each patch (grid-cell) is approximately 3.6 m^2^ (i.e., 1.9 x 1.9 m). The pasture included grass and inedible weeds patches as well as a semi-circular pond of water, approximately 1-acre large, with a constant depth of 0.5 m (Fig. 2). In an actual grazing system, this would be analogous to a well-fed pond that maintains a constant volume as water evaporates and is consumed by cattle. The pasture environment contained trees to provide shade for the cattle. Shade patches were located within a 4–patch radius (R= 7.6 m) around the trees. Patch variables included patch type (grass, weeds, water), concentration of STEC in the patch, and specifically for grass patches, the height of grass, which influenced cattle grazing behavior. Concentration of STEC was in colony forming units (CFU) per patch, which was diluted per L in water patches. The number of contaminated and non-contaminated fecal-pats, as well as the concentration of STEC per patch was tracked per time step (10-minute).

There were three types of agents in the model, including cattle, trees, and patches. Cattle agents moved and engaged in different behaviors, and were colonized by STEC. Tree agents were placed in random, non-water locations in the grid system and did not interact with cattle directly. Their function was to provide shade. Patches were the environment that cattle and trees inhabited and thus did not move, but had several characteristics that were individually assigned, including type (edible grass, inedible weeds, water, shade), grass height (if grass), depth (if water), and contamination status. We assumed a closed cattle population size (N) of 25 to maintain an intensive stocking density of approximately one animal per acre. Population demography, such as birth and death, was not considered in this model due to a relatively short period of simulation (60 days). The cattle specific state variables were related to animal behavior, pathogen exposure history, and epidemiological status of the animals. Behavior state variables represented the social status of the animal (coded as binary, dominant or subordinate cattle), which was used to determine how cattle moved during drinking and resting behaviors. The cattle agents also had epidemiological state variables. Cattle could be categorized into one of the four epidemiological states: susceptible, colonized and in a latent period (without shedding), colonized and shedding, and partially susceptible (after becoming colonized once). Cattle were classified as susceptible to STEC if they had not been colonized with STEC previously. Upon transmission (either direct or indirect), a latent period took place in which cattle were colonized with STEC but did not yet shed the pathogen in feces. Once animals recovered from colonization, they gained partial immunity and remained partially susceptible to re-colonization (Chen et al., 2013a). In addition to the epidemiological states, additional state variables traced the transmission routes (direct or indirect) by which colonized cattle had been exposed.

#### 1.3. Process Overview and Scheduling

Cattle engaged in different activities depending on the time of day and the temperature. All major cattle activities occurred on an hourly basis, while sub-models within those activities (movement, transmission, etc.) occurred on a 10-minute time-step. The schedule of the model, including the sequence of actions and their corresponding sub-models, was shown in Figure 3. Briefly, in each day, cattle slept, grazed on grass, drank from the pond, and rested, either in place or under the shade of trees. During each of these activities, direct transmission of STEC might occur if a shedding individual made contact with a susceptible individual. During drinking and grazing, indirect transmission might occur if cattle ate or drank contaminated graze or water. Cattle defecation occurred at a specified probability (**dailydefavg,** overall average per individual), with equal probability for any individual in all activities. Environmental processes such as decay of STEC, grass growth, and temperatures were updated in each time step (10-minute). On a daily basis (i.e., 144-step), the model recorded the number of cattle newly colonized with STEC from each route (Direct, Water, Graze).

### 2. Design Concepts

### *2.1. Basic Principles*

This model was designed to address the overall question of why and how a higher prevalence of STEC colonization in cattle tends to occur during warmer months than cooler months. It operated on the basic principle that STEC colonization arouse from both direct host-to-host contact and indirect environment-to-host contact. It assessed the hypotheses that STEC incidence increases during warming months relative to cooler months because temperature modulated animal behavior that drove contact structures between cattle, and between cattle and the environment, increasing the overall animal exposure to STEC (Fig. 1). In particular, this model assessed whether increasing temperature drove cattle aggregation and volumes of water consumed, resulting in higher incidence of STEC. In addition, it quantified the relative contribution of direct (host-to-host) and indirect (environment-to-host) transmission routes to overall STEC incidence.

In the model, simple rules governed daily animal activities and movements to generate realistic patterns of animal aggregation and fecal-pat distribution in the environment. Some rules were from direct field observation of grazing cattle at the East Tennessee Research and Education Center - Blount Unit in August 2013 while others were based on existing literature on the topic. During field observations, which generally comported with literature description, cattle behaved synchronously [2], and within a general daily scheme influenced by the time of day and ambient temperature [3,4]. Similarly, cattled in our model followed a diurnal cycle of sleeping, grazing, and drinking, with cattle engaging in particular activities at specific hours of the day (Fig. 3). Of these activities, duration of grazing and resting behavior, and volume of water consumed were dependent upon temperature. First, while cattle in the model always rested for some amount of time during the day, if hourly temperature exceeded a specified threshold during these periods, cattle would spend less time grazing, and more time resting. In addition, resting behavior was also influenced by temperature. If the temperature threshold was not reached during the resting period, cattle simply rested in their current place. However, if the temperature threshold was reached, then cattle moved from their current place and rested under a tree shade as a group. These behaviors reflected the observation that as temperature increases, cattle tend to spend less time grazing and more time resting under shade in groups to relieve heat stress [5–7]. Movement in grazing beef cattle was influenced by their social hierarchy in which dominant individuals guided their group members movements [8,9]. In this model, there was a single dominant individual that subordinated all other individuals when travelling to rest under trees and moving to the pond to drink. While grazing, cattle searched for grass themselves regardless of their social status. Second, cattle in the model consumed a larger volume of water with increases in temperatures, reflecting a similar pattern in seen in grazing systems [10,11].

Cattle tend to move plant material from where they graze to where they rest, drink, and ruminate by defecation [12]. Because cattle colonized with STEC shed pathogens in their feces, this could result in a wide spatial distribution of contaminated substrates. In the model, cattle excreted fecal-pats, contaminated with STEC or otherwise, during all activities except for sleeping. The concentration of STEC in contaminated patches increased with each colonized fecal-pat and decreased with time. Thus, resting under tree shade versus additional grazing and resting in place had implications on direct host-to-host transmission by influencing cattle aggregation and by on grass-to-host transmission by exposure to STEC through grazing. Likewise, increased drinking with temperatures raised the risk of water-to-host transmission of STEC*.*

#### 2.2 Emergence

The most significant emergent model output was the count of incident cases (e.g. number of new colonizations) per day, which could then be summed over the course of the simulation period (60 days). Another emergent output was the average number of colonizations caused by the index case in an otherwise susceptible cattle population, otherwise referred to as the basic reproduction number. To calculate the basic reproduction number, the number of new colonizations produced by the initially infected individual over the duration of their first colonization period (either via direct or indirect pathways) was recorded per simulation of a given model parameter. Then, these counts were averaged over multiple simulations. Cattle movement and behaviors are not emergent outcomes as they are imposed by the model rules. However, the distribution of manure emerged from the model as a byproduct of model activities.

#### 2.3 Sensing

In the model, cattle were assumed to know the value of certain variables and were able to sense others. The cattle knew their social status (dominant/subordinate), the time of the day, the activity to engage in based on the time of day and temperature, the ambient temperature, plant edibility (grass or of weeds), and patch type (plant, shade,, or water) patches. Cattle were able to sense grass and water from a certain distance of themselves, and they were able to sense the social status of other cattle (dominant vs subordinate individuals). Uncertainty in sensing is not considered explicitly, but uncertainty in behavior was incorporated through probabilistic hurdles. For example, during grazing times, cattle had a certain probability of staying and eating on a patch versus moving to other grass patches. This method simulated variability in individual decision-making.

#### 2.4 Interaction

Cattle interacted among themselves and with the environment. The dominant individual influenced the movement of the herd during drinking or resting, and cattle could interact with each other when in close proximity. When these contacts occurred, colonized and shedding individuals transferred STEC to non-colonized individuals. Cattle consumed water when drinking in a water patch, ate grass when grazing in a grass patch, and ingested STEC when either was contaminated. Water in the pond was assumed to remain a constant volume, while grass height was reduced with grazing. If grass height was grazed below a certain threshold, cattle could not graze on it until it regrew. Cattle defecated while grazing, drinking, resting, and sleeping. Shedding cattle increased the concentration of STEC on patches they contacted by defecating contaminated feces onto them. Tree agents did not interact with other agents or the environment; trees aided only in visualization of the model environment and were centered in the shade patches. Cattle agents were attracted to shade patches during resting periods.

#### 2.5 Stochasticity

The initial locations of grass patches, weed patches, and cattle were set randomly. Sub-models describing cattle interactions (both among cattle themselves with the environment), exposure, and STEC colonization were also stochastic (see sub-models section).

#### 2.6. Observation

To address the primary study questions, the count of incidence cases and sources of transmission (direct, indirect) in the herd was recorded on a daily basis. To observe the implicit dynamics of this model, hourly ambient air and water temperatures, average survival rates of STEC in water and soil, average grass height, and average fecal-pats produced per day per individual were captured by the model as well.

#### 2.7. Collectives

Aggregation of agents was not included in this model.

#### 3. Modelling Details

##### **3.1 Initialization**

The environment was initialized at the beginning of each simulation (S1 Text, Fig. 1). Water patches were located in a semi-circle central to the left edge of the pasture creating a 1-acre pond. Grass (edible) and weed (inedible) patches were assigned randomly in space while maintaining approximately a 4:1 grass-weed ratio. Shade patches were set up in a 4-patch radius (8 m) around tree agents. Tree number (N_T_) was set to five and these trees were created during initialization and were located in random, non-water positions across the pasture. Twenty-five (25) cattle agents were created and placed at random locations, with one dominant (visually larger than the submissive cattle) individual. One individual was randomly assigned as colonized and shedding at the beginning of the simulation to start a potential epidemic, and was independent of its social status.

**S1.3, Fig. 1. NetLogo model environment**


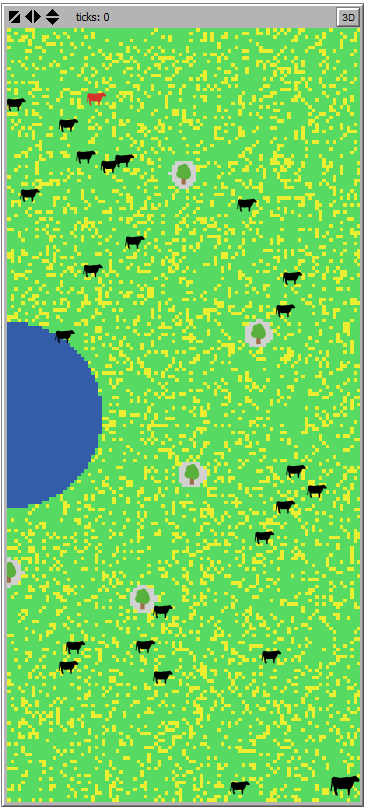


The NetLogo model environment consisted of a 101 x 221 grid pasture (1.9 m per side (3.6 m^2^)), containing grass (green), weeds (yellow), a 1-acre pond (blue), 25 cattle (black, larger size icon indicated the dominant individual), and five trees (with tree symbol). The initially infected individual was displayed in red while all other susceptible cattle were displayed in black. Note, for illustrative purposes, cattle graphics shown above are much larger than life-size.

##### **3.2 Input data**

Temperature data (daily maximum and minimum temperature) were acquired from the National Climate Data Center (NCDC, 2013) for McGhee Tyson Airport station (station ID: GHCND:USW00013891) in Knoxville, Tennessee, the closest weather station to the observation field (approx. 8 km). The temperature data were converted to hourly air and water temperatures (as described below), and were used to estimate STEC decay rates in water and manure, and dictate cattle behavior. Temperature data are available in S4 Folder.

### *3.3 Sub-models*

*Temperature Calculation*. This sub-model used daily maximum and minimum temperature in a modified version of a sine-wave model developed by [13] to interpolate the hourly ambient temperature and reconstruct diurnal temperature fluctuation (eq. 1, 2,3): ${T(hrsec)}_{0-4}= \left( \frac{MAX2-MIN2}{2} \right)\cdot\cos\left\lceil\left( hrsec-9 \right)\cdot\frac{180}{14} \right\rceil+\frac{MAX2+MIN2}{2}$(1)

${T(hrsec)}_{5-15}= -\left( \frac{MAX1-MIN1}{2} \right)\cdot\cos\left( hrsec\cdot\frac{180}{10} \right)+ \frac{MAX1+MIN1}{2}$(2)

${T(hrsec)}_{15-23}= \left( \frac{MAX1-MIN2}{2} \right)\cdot\cos\left\lceil\left( hrsec-15 \right)\cdot\frac{180}{14} \right\rceil+\frac{MAX1+MIN2}{2}$ (3)

In these equations, the subscript of T(hrsec) describes the continuous (i.e. time-step based) temperatures for that hour range; for example 0-4, T(hr)_0-4_ was the continuous temperature for hours 0-4. *MAX1* was the daily maximum temperature, *MIN1* was the daily minimum temperature, *MIN2* was the minimum temperature of the next day, and *MAX2* was the daily maximum temperature from the previous day.

Water temperature was approximated as a function of ambient air temperature according to the following equation [14],

$T_{w}= a+ \frac{a}{1+e^{c(d-T_{a})}}$ (4)

where *T_w_* was estimated water temperature, *a* was minimum water temperature, *b* was maximum water temperature, *c* was a measure of the steepest slope of the *T_w_* function (when plotted against *T_a_*, ambient temperature) and *d* was the air temperature at the inflection point. Parameter values were gathered from a literature source [15], and we assumed a homogeneous temperature across the pond because the pond was shallow (0.5 m deep).

*Cattle activities.* Cattle activity sub-models were intended to simulate realistic cattle behavior and movement, and included 4 major sub-models for grazing, drinking, resting, and sleeping. The time in each sub-model was based on the hour of the day, with time for grazing and resting also determined by ambient air temperature (Fig. 3). In all but the sleep sub-model, cattle moved to or towards particular patch types or towards the dominant individual, depending on the activity. To incorporate stochasticity into these movements, a general “random-movement” sub-model was invoked (with varying probability by other sub-models) that determined a random distance (Poisson distributed with an average of 5 cells) and direction (within 90° of current direction) for cattle to move.

*Grazing*. The grazing sub-model was always activated from hours 5 through 7, and hours 21 through 22. When ambient air temperature was less than the specified temperature threshold (**T_thr_**), cattle also grazed during hours 10 and 15. Grazing activity was not influenced by their social status. Instead, if an individual was at a patch with edible grass, there was a probability ***p_movenewpatch_*** that the individual would search for grass in a new patch or stay where it was and graze. If the former, it would search for an edible patch within 1 patch of itself and within 45°C of its “cone of vision”. If no such patch was found, then the individual moved to a random patch using the “random-movement” sub-model_._ The result of these rules simulated how cattle spread out throughout a pasture while grazing. When grazing, cattle would eat between 2 and 3 units of grass height out of a maximum possible of 5. Cattle would not starting eating in a patch with less than 1 unit height of grass, though they could reduce the height to less than 1 in a grazing bout. The total grass consumed during a grazing bout was used to compute the total uptake of *E.coli* (see “Transmission sub-models: grazing” below) for that grazomg bout.

*Resting*. Cattle resting time and resting behavior differed depending on the temperature. The resting sub-model was activated from hours 10 to 12, and from hours 15 to 17. If ambient temperature was greater than ***T_thr_*** at hours 10 and 15, cattle would move to rest under trees in shades. If the temperature was less or equal to **T_thr_**, cattle would continue to graze for an additional hour. If the hourly ambient temperature was less than or equal to **T_thr_** at hours 11-12, and hours 16-17, cattle would rest in place, with a small probability (***p*_movewhilerestsub_** ) of moving during the rest period. If the temperature was greater than **T_thr_** , cattle would either move to or continue resting under trees in the shade. Shade resting behavior was influenced by the dominant individual. At the beginning of each resting hour, the dominant individual randomly selected a shade-patch at random. If shade resting conditions were met, the dominant individual began moving towards the shade patch, and subordinate cattle moved towards the dominant individual at a random, slightly offset angle (30° ± 15), with the goal of getting either in the shade or being within a short distance of the dominant individual. Once in the shade, the dominant individual had a small probability of moving 2 spaces at random (***p_movewhilerest_dom_***), and subordinate cattle had a small probability of following the dominant individual if it moved away from the shade (**p_movewhilerest_sub_**).

*Drinking.* The drinking sub-model was activated from hours 8 through 9, 13 through 14, and 18 through 19. Once activated, the dominant individual moved towards the closest water patch with probability ***p_nearestpatch_***, and to another random water patch otherwise. Subordinate cattle moved towards the dominant individual in a similar manner as described above for resting under trees. Once in a water patch, the dominant individual either stayed where it was with probability ***p_stayanddrink_***, or moved randomly to another patch according to the move-random submodel. Similarly, subordinate cattle either stayed in the water patch they were in, or moved with probability ***p_movetodominant_*** towards the dominant individual. While in a water patch, cattle drank a volume of water (L) that was determined by the daily average temperature (arithmetic mean of daily maximum and minimum temperature, for simplicity) based a non-linear function

$Liters=33.51213-0.74978*avg daily temp+0.05806{*avg daily temp}^{2}$(5)

derived from data presented by Parish and Rhinehart (2008) of approximate daily water intake by 800-1000 lb (362-453 kg) finishing beef cattle. The total liters of water consumed per drinking bout was used to compute STEC consumed (see “Transmission sub-models: Drinking” below). Total liters drinking per day was based on an expected average per day, adjusted for total number of minutes spent in drinking. Since cattle had to move to water patches from grazing or resting activities, the number of minutes spent drinking per day was adjusted from 36 (6 hours * 6 ticks per hour) to 30, to account for travel time to the lake.

*Sleeping.* When sleeping, cattle were assumed to be stationary in their patch and did not graze. However, infectious cattle could transmit STEC directly to susceptible cattle within the transmission direct transmission distance threshold (***ddt****)* (see “Transmission sub-models: Direct”), and defecate.

*Epidemiological Sub-models.* There were five epidemiological sub-models, including functions that governed shedding of contaminated feces (Shed), uptake of STEC (Transmission), STEC colonization (Colonize), updating of cattle epidemiological status (Update-cows), and updating of STEC on patches (Patches-update). Of these, Colonize only occurred at hour 0 when the cattle were sleeping, while the others occurred in each activity at each time-step after the completion of the activity.

*Shed sub-model.* The shed sub-model kept track of defecations per individual, and governed how STEC was added to water and grass patches. Daily average defecation (approximate 2000 g fecal pats per day) was not based on the units of grass consumed, but was instead based on a constant dry matter intake per hour of grazing [16], calculated as:

$M_{kg}=DMI*4.158-BW*0.0246$ (6)

Where M_kg_ was the kg weight of manure produced (kg) per day per animal, DMI was dry matter intake, and BW was body weight (assumed an average of 437 kg, (Nennich et al., 2005; within the range of body weight assumed for the drinking function above)). Because body weight in this equation had a small effect relative to DMI, it was held constant. Dry matter intake, however, varied with the amount of time grazing (0.026 kg/min) adapted from [17], with time spent grazing varied based upon temperature. As noted above (Grazing and Resting sub-models), grazing and resting occurring in hours 10 (morning) and 15 (afternoon) depended on whether the ambient temperature exceeded **T_thr_** (as noted above in the Grazing and Resting sub-models). Average defecation (number of fecal pats) per expected day^-1^per day) depended on whether **T_thr_** was reached in both the morning and afternoon resting periods (11 fecal-pats day^-1^), whether it was reached in the afternoon but not the morning (14.3 fecal-pats day^-1^), or it was not reached at all (17.6 fecal-pats day^-1^). The probability of defecation was constant, and was calculated as the average defecation expected for the day, divided by the the total number of time steps per day (10-munite per step, a total of 144 steps per day).

Colonized cattle began shedding STEC after the latency period (***latent_phase_***). When an infectious fecal-pat was deposited in a patch, the identification number of the depositing individual was recorded in that patch, and the pathogen amount increased by **C**_cowpat_ CFUs in the patch. This value was the product of the number of CFUs per gram of fecal-pat (**C**) (determined through calibration) and the average mass of a fecal-pat (**avg_mass**) [18]. In a water patch, this concentration was assumed to be homogeneous through the volume of water (1.8 m^3^ (0.5 m depth * 1.9 m * 1.9 m)).

*Transmission sub-models.* Three transmission sub-models governed the quantity of STEC that cattle were exposed to, through direct and indirect transmission pathways. When transmission was through the direct pathway, the identification number of the contributing infectious individual was recorded. When STEC was taken up by cattle through grazing or drinking, the identification number of the individual who deposit a contaminated fecal-pat in the patch was associated as the source of STEC for that exposure. Because multiple infectious individuals can make infectious source ambiguous, this information was only used in calculating R0, as the likelihood of a single source of infection during the initially colonized individual’s infectious period (particularly for indirect pathways) was relatively high. The total STEC accumulated throughout a day (144 steps) and was evaluated in the colonization sub-model (see below) to determine colonization status.

*Transmission sub-model: direct.* In this sub-model, a susceptible individual was exposed to STEC from a colonized individual if they were less than the direct transmission distance threshold (***ddt****)* from them. For each time-step within the distance threshold, the quantity of CFUs was randomly drawn from a Poisson-lognormal distribution with mean and standard deviation (**pln_mean_, pln_sd_**) determined via calibration.

*Transmission sub-model: grazing*. In this sub-model, STEC exposure was through grazing, and was proportional to the product of the STEC concentration in the patch, the amount of grass eaten, and a probability (***p_GrazeInfect_****)* of encountering STEC while grazing. This was calculated as:

$STECexposed=Patch CFUs* \left( grass units consumed | pregraze units available \right)* p_{GrazeInfect}$ (7)

Cattle generally avoid grazing around a fecal-pat [19]. Thus, the contact probability between STEC in the fecal-pat and the individual was lowered with ***p_GrazeInfect_*** to account for this avoidance behavior.

*Transmission sub-model: drinking.* Within a water patch, STEC concentration was assumed to be homogeneous. Therefore, the amount of STEC taken up by drinking was simply the product of the volume of water (L) consumed and the concentration (CFU/L) of STEC in water patch.

*Colonization sub-model.* This sub-model was evaluated at hour 0 each day, and used the total STEC accumulated over the previous day in using a simple dose-response function (Chen et al., 2013a) to determine the probability of colonization, ***p_col_* _:_**

$p_{col}=\frac{1}{1+K/{CFU}}$ (8)

In this equation, ***K*** was the dose required to colonize 50% of a population. This equation is a re-arranged form the Hill-1 Dose response equation presented in [21]. The sub-model assumed that an individual that had previously recovered from STEC (see update-cow sub-model) gained partial immunity, thus increasing ***K*** by a factor of ***SImult***. Therefore, the minimum colonization dose (***K***) was assumed to be higher for previously recovered individuals (**K** * **SImult**). If colonization occurred, the transmission pathway contributing the majority of STEC for that colonization event was recorded, and cattle colonization status changed from “not colonized” to a “colonized” epidemiological state. If a colonization did not occur, the total STEC accumulated from the previous day was cleared and reset to 0.

*Update-cows sub-model.* This sub-model kept track of epidemiological states of cattle and time since colonization. Once ***latent_phase_*** had elapsed, cattle were assigned to “infectious” (i.e. shedding) status, at which point they began excreting STEC, and thus were capable of infecting other cattle or contaminating the environment. Once shedding cattle completed the infectious period, they were assigned to recovered state. Cattle had a fixed recovery rate **γ** (inverse of infectious period) once colonized.

*Update-patches sub-model.* This sub-model updated patch conditions, including grass re-growth and the decay of STEC in the patch. Grass had a fixed growth rate (*α*) of 10^-3^ unit/hour (assumed), but did not grow above 5 height units. STEC decayed exponentially in the environment, with a first-order decay rate. Because temperature drives pathogen growth and death in the environment, a *Q_10_* model was used to further adjust the decay rate based on temperature [22,23]. The *Q_10_* model is a modified version of the Arrhenius equation used to estimate the changes in the decay rate as function of temperature,

$k_{T}=k_{r}{Q_{10}}^{\frac{T-Tr}{10}}$ (9)

where k_T_ was the bacterial decay rate at given temperature *T* (°C), *k_r_* was the bacterial decay rate at the reference temperature (***Tr***), and Q_10_ was the temperature coefficient that gave the rate of change for each temperature increase of 10 °C. Although the function assumed that bacteria decay increased with temperature in general, decay occurred faster in aquatic environments due to increased competition for resources, particularly in organically enriched water like agricultural ponds. To reflect this, parameters for the *Q_10_* model for water (k_r_ = 0.388 and *Q_10_* =1.548) and manure (k_r_ = 0.042 and *Q_10_* =1.48) were based on estimates selected from those presented in Blaustein et al. (2013) and Martinez et al. (2013), respectively, in which decay rate for water (in agricultural settings) was higher than manure. The reference temperature for Q_10_ models was 20 °C.

**References Cited**

1. Wilensky U. NetLogo [Internet]. Evanston, IL; 1999. Available: http://ccl.northwestern.edu/netlogo/.

2. Stoye S, Porter MA, Stamp M. Synchronized lying in cattle in relation to time of day. Livest Sci. Elsevier; 2012;149: 70–73. doi:10.1016/j.livsci.2012.06.028

3. Kilgour RJ, Uetake K, Ishiwata T, Melville GJ. The behaviour of beef cattle at pasture. Appl Anim Behav Sci. Elsevier B.V.; 2012;138: 12–17. doi:10.1016/j.applanim.2011.12.001

4. Ruckebusch Y, Bueno L. An analysis of ingestive behaviour under field conditions and activity of cattle. Appl Anim Ethol. 1978;4: 301–313.

5. Rosselle L, Permentier L, Verbeke G, Driessen B, Geers R. Interactions between climatological variables and sheltering behavior of pastoral beef cattle during sunny weather in a temperate climate. J Anim Sci. 2013;91: 943–949. doi:10.2527/jas.2012-5415

6. Zuo HT, Miller-Goodman MS. Landscape use by cattle affected by pasture developments and season. J Range Manag. 2004;57: 426–434 ST–Landscape use by cattle affected by. doi:10.2307/4003970

7. Blackshaw JK, Blackshaw AW. Heat stress in cattle and the effect of shade on production and behaviour: A review. Aust J Exp Agric. 1994;34: 285–295. doi:10.1071/EA9940285

8. Radka S, Spinka M, Arias JL, Sime P. Graded leadership by dominant animals in a herd of female beef cattle on pasture. Anim Behav. 2010;79: 1037–1045. doi:10.1016/j.anbehav.2010.01.019

9. Arave CW, Albright JL. Cattle Behavior. J Dairy Sci. 1981;64: 1318:1329.

10. Hoffmann I. Climate change and the characterization, breeding and conservation of animal genetic resources. Anim Genet. 2010;41: 32–46. doi:10.1111/j.1365-2052.2010.02043.x

11. Parish J, Rhinehart J. Beef Cattle Water Requirements and Source Management. Ext Serv Mississippi State Univ. 2008; 1–8. Available: http://msucares.com/pubs/publications/p2490.pdf

12. Flack S. The art and science of grazing. White River Junction, VM: Cheslea Green Publishing; 2016.

13. Schaub WR. A method for estimating missing hourly temperatures using daily maximum and minimum temperatures. 1991;7: 157–164.

14. Mohseni O, Stefan HG, Erickson TR. A nonlinear regression model for weekday stream temperatures. Water Resour Res. 1998;34: 2685–2692.

15. Morrill JC, Bales RC, Asce M, Conklin MH. Estimating Stream Temperature from Air Temperature : Implications for Future Water Quality. 2005;131: 139–146.

16. Nennich TD, Harrison JH, VanWieringen LM, Meyer D, Heinrichs AJ, Weiss WP, et al. Prediction of manure and nutrient excretion from dairy cattle. J Dairy Sci. Elsevier; 2005;88: 3721–33. doi:10.3168/jds.S0022-0302(05)73058-7

17. Hamilton SA, Kallenbach RL. Managing Pasture for Yield, Quality, Persistence and Intake [Internet]. Extension Service of University of Missouri. 2015. Available: http://extension.missouri.edu/p/M185

18. Muirhead RW, Littlejohn RP. Die-off of Escherichia coli in intact and disrupted cowpats. Soil Use Manag. 2009;25: 389–394. doi:10.1111/j.1475-2743.2009.00239.x

19. Phillips C. Social Behaviour. Cattle Behavior and Welfare. 2nd ed. Wiley-Blackwell; 2002. pp. 84–122.

20. Chen S, Sanderson M, Lanzas C. Investigating effects of between- and within-host variability on Escherichia coli O157 shedding pattern and transmission. Prev Vet Med. Elsevier B.V.; 2013;109: 47–57. doi:10.1016/j.prevetmed.2012.09.012

21. Brouwer AF, Weir MH, Eisenberg MC, Meza R, Eisenberg JNS. Dose-response relationships for environmentally mediated infectious disease transmission models. PLoS Comput Biol. 2017;13: e1005481.

22. Blaustein RA, Pachepsky Y, Hill RL, Shelton DR, Whelan G. Escherichia coli survival in waters: Temperature dependence. Water Res. Elsevier Ltd; 2013;47: 569–578. doi:10.1016/j.watres.2012.10.027

23. Martinez G, Pachepsky YA, Shelton DR, Whelan G, Zepp R, Molina M, et al. Using the Q10 model to simulate E. coli survival in cowpats on grazing lands. Environ Int. Elsevier Ltd; 2013;54: 1–10. doi:10.1016/j.envint.2012.12.013
