# Supplementary material for: Investigating behavioral drivers of seasonal Shiga-Toxigenic Escherichia Coli (STEC) patterns in grazing cattle using an agent-based model
Source: PLoS One. 2018 Oct 10;13(10):e0205418. doi: 10.1371/journal.pone.0205418 (PMC6179278; doi:10.1371/journal.pone.0205418)
Supplement: S2 Text — (DOCX) [file pone.0205418.s002.docx]

# **S2.1 Latin Hypercube-based global sensitivity analysis to produce the calibrated model**

**S2 Text, Table 1. Partial Rank Correlation Coefficients (PRCC) of global Latin hypercube sensitivity analysis**

|  |  |  |  | 95% CI | |
| --- | --- | --- | --- | --- | --- |
| Temperature (°C) | Parameter | PRCC | bias | Lower | Upper |
| 20 | ddt | 0.139 | -0.003 | 0.071 | 0.205 |
|  | **P_grassinfect_** | 0.650 | 0.001 | 0.617 | 0.681 |
|  | **ᵞ** | 0.205 | 0.002 | 0.136 | 0.264 |
|  | **pln_mean_** | 0.276 | 0.001 | 0.206 | 0.347 |
|  | **C** | 0.668 | 0.000 | 0.636 | 0.706 |
|  | **K** | -0.756 | -0.001 | -0.780 | -0.734 |
|  | **SI_mult_** | -0.076 | -0.003 | -0.127 | -0.009 |
| 24 | ddt | 0.160 | 0.003 | 0.102 | 0.224 |
|  | **P_grassinfect_** | 0.609 | 0.004 | 0.563 | 0.652 |
|  | **ᵞ** | 0.204 | 0.002 | 0.148 | 0.259 |
|  | **pln_mean_** | 0.242 | -0.003 | 0.171 | 0.305 |
|  | **C** | 0.643 | 0.000 | 0.605 | 0.684 |
|  | **K** | -0.741 | -0.002 | -0.775 | -0.711 |
|  | **SI_mult_** | -0.071 | -0.004 | -0.122 | 0.002 |
| 25 | ddt | 0.476 | 0.001 | 0.435 | 0.523 |
|  | **P_grassinfect_** | 0.318 | 0.000 | 0.267 | 0.366 |
|  | **ᵞ** | 0.224 | -0.003 | 0.161 | 0.286 |
|  | **pln_mean_** | 0.607 | -0.003 | 0.566 | 0.651 |
|  | **C** | 0.359 | 0.004 | 0.296 | 0.412 |
|  | **K** | -0.647 | -0.003 | -0.683 | -0.602 |
|  | **SI_mult_** | -0.080 | 0.005 | -0.139 | -0.017 |
| 30 | ddt | 0.473 | 0.004 | 0.425 | 0.530 |
|  | **P_grassinfect_** | 0.288 | 0.000 | 0.228 | 0.353 |
|  | **ᵞ** | 0.191 | 0.002 | 0.138 | 0.238 |
|  | **pln_mean_** | 0.621 | 0.000 | 0.579 | 0.664 |
|  | **C** | 0.379 | 0.000 | 0.322 | 0.433 |
|  | **K** | -0.643 | 0.000 | -0.679 | -0.602 |
|  | **SI_mult_** | -0.082 | -0.001 | -0.150 | -0.007 |

95% CIs of partial rank correlation coefficients (PRCC) of simulations parameterized with 1000 unique parameter sets of 7 variables derived from a Latin Hypercube sampling-based global sensitivity analysis. Simulations run at constant 20°C, 24°C, 25°C, and 30°C to differentiate temperature-threshold versus continuous temperature effects. PRCCs at 24°C and 25°C were found to be similar to PRCC’s at 20 and 30 respectively. Parameters include: **ddt** = distance of direct contact; **pln_mean_** = mean of the Poisson-log normal distribution (sampled for direct transmissions), **C** = STEC concentration in cow pats; **P_grassinfect_** = proportion of CFU’s up taken per contaminated grass unit, per grass patch when grazing; **K** = median population dose of STEC expected to result in colonization; **ᵞ** = recovery time (days); and **SI_mult_** = amount **K** multiplied by in the case of secondary colonizations.

**S2.2. Sensitivity to structural characteristics of model environment and cattle density**

To assess the sensitivity of the calibrated model to structural characteristics of the model environment and cattle density, a sensitivity analysis was conducted using a partially Latin Hyper Cube-sampling design. The model was simulated at two constant temperatures (20°C and 30°C) using 1000 unique parameter over 5 numeric variables: the number of trees (1-10), the shade radius of trees (2-8 meters), the grass to weed ratio (0.1-1), lake size (0-0.82 ha (0-2 acres)), cattle density (10-50). In addition, lake position and shape was randomly sampled 1000 times from 1 of 9 positions, including semi-circles on the edges of the sides (2 (as in the factorial model simulations) and long-ends of the pasture (2), wedge-shaped positions in each corner (4), and a circular shape in the middle of the pasture

PRCC analysis found that of numeric variables included, only the number of cattle and the grass to weed ratio had more than weak influences (|PRCC| < 0.1) on the count of incident cases (S2 Text, Table 2). In both cases, these variables were positively correlated with the count of incident cases, with the effect of the number of cattle larger at 30°C, and effect of grass to weed ratio larger at 20°C. The effect of increasing density would be expected, as increasing cattle would provide more opportunity for both direct transmission when clustering under trees to rest is forced, and indirect transmission through the production of additional manure. In the case of the grass to weed ratio, this effect was because cattle had to move less to find edible grass to graze, resulting in grazing more often in previously grazed locations, and therefore more exposure to contaminated grass. Examining counts of incident cases by position revealed that while the total number of incident cases was higher at 30°C than 20°C (S2 Text, Fig 1), and the number of graze-based (S2 Text, Fig 2) and direct-based cases (S2 Text, Fig 3) was highest at 20°C and 30°C, respectively, more counts tended to occurred when the lake was positioned along the corners of the pasture regardless of temperature. For direct-driven transmission, this is likely because positioning the lake in the corner of the rectangular pasture encouraged more clustering during drinking relative to a semi-circle position, allowing more direct transmission to occur. For graze-driven transmission, this is likely because cattle tended to cluster on one end of the pasture over the day to be near water, promoting clustering of contaminated manure.

**S2 Text, Table 2. PRCCs of sensitivity analysis of environmental variables**

| Temperature | Variable | PRCC | bias | 95% CI | |
| --- | --- | --- | --- | --- | --- |
|  |  |  |  | Lower | Upper |
| 20°C | Number of Trees | -0.038 | 0.031 | -0.099 | 0.022 |
|  | Shade radius of trees | 0.035 | 0.032 | -0.026 | 0.097 |
|  | Grass to weed ratio | 0.274 | 0.030 | 0.214 | 0.335 |
|  | Size of Lake | 0.031 | 0.032 | -0.033 | 0.094 |
|  | Number of cattle | 0.343 | 0.029 | 0.285 | 0.399 |
| 30°C | Number of Trees | 0.063 | 0.027 | -0.011 | 0.097 |
|  | Shade radius of trees | -0.120 | 0.028 | -0.170 | -0.065 |
|  | Grass to weed ratio | 0.078 | 0.033 | 0.017 | 0.150 |
|  | Size of Lake | 0.018 | 0.037 | -0.066 | 0.093 |
|  | Number of cattle | 0.442 | 0.025 | 0.396 | 0.499 |

PRCC values for the influence of environmental structure variables on total incidence counts produced by the calibrated model. Number of trees (1-10); Tree shade radius (2-8 m); Grass to weeds ratio (0.1-1); Lake size (0-0.82 ha (0-2 ac)), and number of cattle (10-50).

**S2 Text, Figure 1. Total incident cases of LHS simulations of environmental variables by temperature and position of lake.**


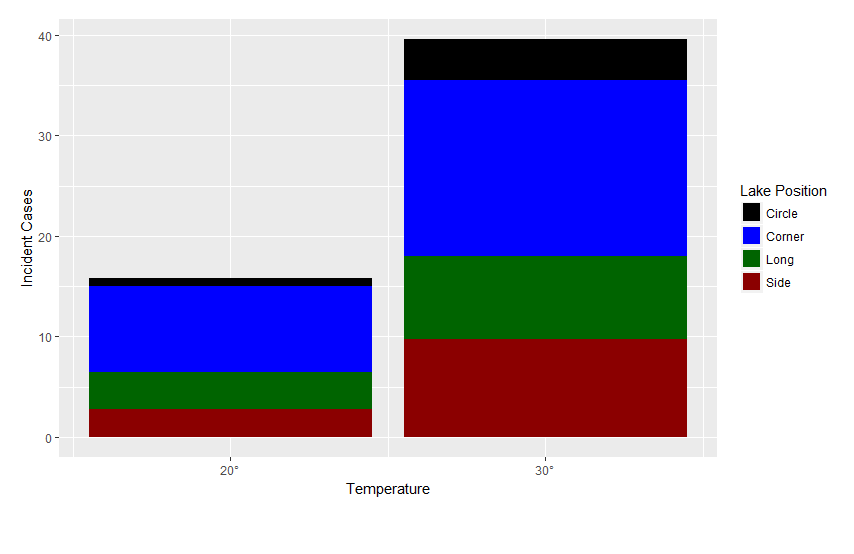


Lake positions included center, corner, side, or long end of the rectangular pasture.

**S2 Text, Figure 2. Graze-based incident cases of LHS simulations of environmental variables by temperature and position of lake.**


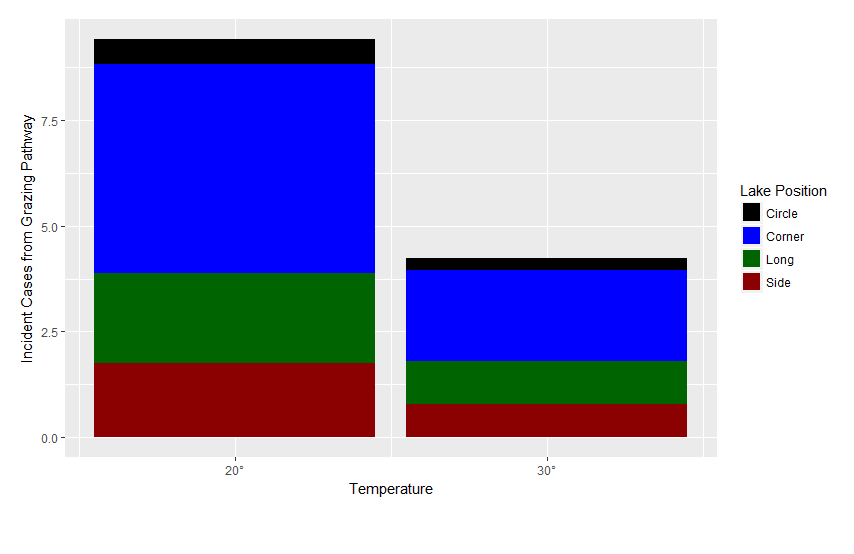


Lake positions included center, corner, side, or long end of the rectangular pasture.

**S2 Text, Figure 3. Direct-based incident cases of LHS simulations of environmental variables by temperature and position of lake.**


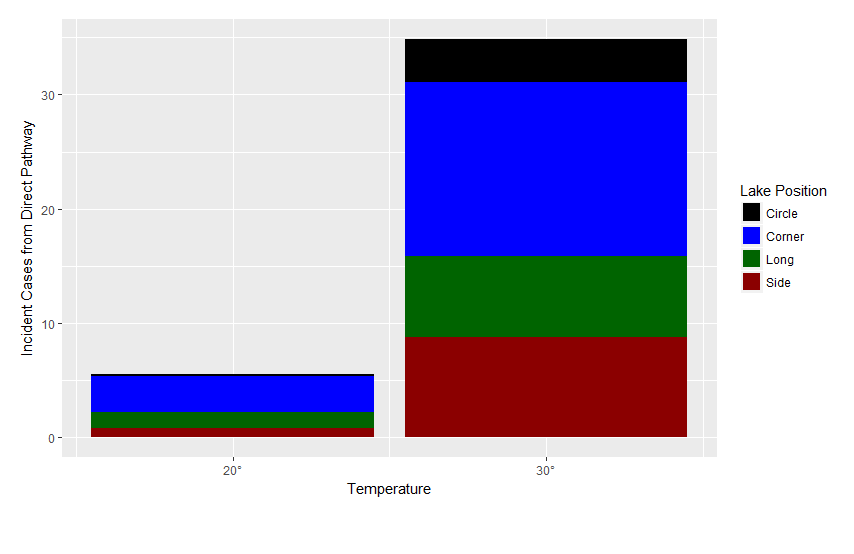
Lake positions included center, corner, side, or long end of the rectangular pasture.
